# Integrin CD11b provides a new marker of pre-germinal center IgA^+^ B cells in murine Peyer’s patches

**DOI:** 10.1093/intimm/dxab113

**Published:** 2021-12-31

**Authors:** Peng Gao, Takahiro Adachi, Shinsaku Okai, Naoki Morita, Daisuke Kitamura, Reiko Shinkura

**Affiliations:** 1 Institute for Quantitative Biosciences, University of Tokyo, Bunkyo-ku, Tokyo 113-0032, Japan; 2 Graduate School of Frontier Science, University of Tokyo, Kashiwa-shi, Chiba 277-8561, Japan; 3 Department of Precision Health, Medical Research Institute, Tokyo Medical and Dental University, Chiyoda-ku, Tokyo 101-0062, Japan; 4 Department of Applied Immunology, Nara Institute of Science and Technology, Ikoma, Nara 630-0192, Japan; 5 Division of Cancer Biology, Research Institute for Biomedical Sciences (RIBS), Tokyo University of Science, Noda, Chiba 278-0022, Japan; 6 Collaborative Research Institute for Innovative Microbiology, University of Tokyo, Bunkyo-ku, Tokyo 113-0032, Japan

**Keywords:** affinity maturation, B cell stimulation, CD11b, pre-GC B cell marker

## Abstract

Activated B cells can enter germinal centers (GCs) for affinity maturation to produce high-affinity antibodies. However, which activated B cells will enter GCs remains unknown. Here, we found a small population of CD11b^+^IgA^+^ B cells located outside of GCs in murine Peyer’s patches (PPs). After injection of the CD11b^+^IgA^+^ PP B cells into a PP of a recipient mouse, they entered GCs forty hours later. They expressed GC surface markers and pre-GC B cell genes, suggesting that CD11b provides a novel surface marker of pre-GC IgA^+^ B cells in murine PPs. Furthermore, independently of dendritic cell activation, CD11b expression on B cells can be induced by bacterial antigens, such as pam3CSK4 and heat-killed *Escherichia coli in vitro.* In addition, mice orally administered with pam3CSK4 or heat-killed *E. coli* increased the number of PP GC B cells within two days, and enhanced the mucosal antigen-specific IgA response. Our results demonstrate that the induction of CD11b on B cells is a promising marker for selecting an effective mucosal vaccine adjuvant.

## Teaser

A crucial role for CD11b in germinal-center reactions

## Graphical Abstract

**Graphical Abstract:**
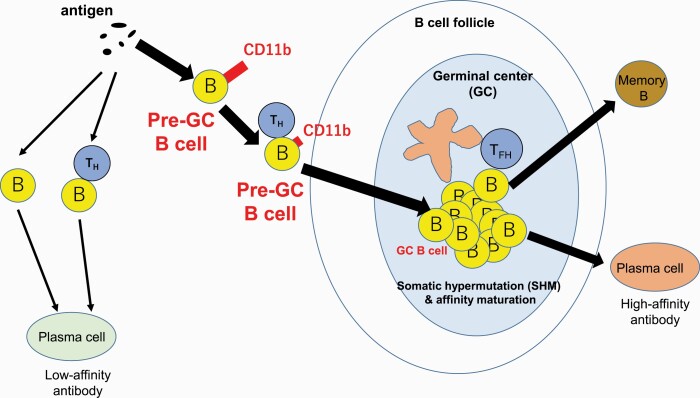

## Introduction

Research into the behavior of B cells and germinal centers (GCs) during immune responses has been motivated by the potential benefit to vaccine development (1, 2). One of the critical criteria for evaluating the effectiveness of a given vaccine is whether it efficiently induces a GC reaction thereby generating high-affinity long-lived plasma cells (PCs) and memory B cells to protect the host from invading pathogens (1, 2). During the GC reaction, B cells follow three stages, pre-GC, GC and post-GC stages (3–5). Upon antigen stimulation, the newly activated naive B cells and the activated memory B cells either directly differentiate into extrafollicular short-lived PCs or migrate to the interfollicular (IF) area to establish a stable interaction with the activated T cells (3–8). After T-B cell interaction, some B cells differentiate into low-affinity PCs, while the others, the pre-GC B cells, migrate into GCs (3, 4). These pre-GC B cells first migrate into the GC dark zone (DZ) and develop into GC B cells, which then proliferate fast and undergo somatic hypermutation (SHM) changing their B cell receptor (BCR) affinity to bind to its specific antigen (3, 4). The B cells with mutations in their immunoglobulin (Ig) genes then migrate from the DZ to the light zone (LZ), where affinity selection occurs with the help of T follicular helper (T_FH_) cells (3, 4, 8). B cells with a high-affinity BCR against their antigen are selected as post-GC B cells to exit the GCs and finally differentiate into long-lived PCs or memory B cells, whereas unselected B cells can return to the GC DZ to accumulate additional SHM for a higher affinity BCR (7).

To initiate the GC reaction, efficient activation of dendritic cells (DCs) has been reported to be essential (3). Therefore, most previous studies focus on selecting the most appropriate adjuvant to stimulate DCs. However, Lycke and co-workers have demonstrated that Peyer’s patch (PP) B cells can sample antigen from microfold (M) cells and further migrate into GCs in mice depleted of DCs (9), indicating that DC activation is not necessary for GC entry. Even though DCs are properly primed, only a small population of activated B cells can develop into pre-GC B cells and enter the GC (4, 6). Recent studies have reported that a transcription factor essential for GC formation, *irf4*, is highly expressed in pre-GC B cells (10, 11). However, there is no known specific surface marker for pre-GC B cells. Hence the criteria for pre-GC B cells, and which kinds of activated B cells enter GCs to produce a high affinity BCR against a specific antigen are not well understood.

To identify the pre-GC B cells, we focused on PPs since the GCs in PPs are constitutively active, so that we can find pre-GC B cells that are entering the existing GCs at any time point (7). We hypothesized that integrin CD11b may be a candidate marker for pre-GC IgA^+^ B cells in PPs. CD11b, the 165-kDa integrin alpha M, associates with CD18 to form the heterodimeric integrin known as macrophage-1 antigen (Mac-1) (12). CD11b is widely considered as a marker for myeloid cells and is known to be involved in cell migration and adhesion (12). In addition, previous studies demonstrated a complex function of CD11b on B cells (13, 14). Ding *et al.* have shown that CD11b negatively regulates BCR signaling to maintain autoreactive B cell tolerance (13). Kunisawa *et al.* demonstrated that CD11b is an early-phase marker of IgA^+^ PCs in murine intestinal lamina propria (LP) and that the structure of PPs is essential for the production of CD11b^+^IgA^+^ PCs (15). These studies motivated us toward investigation of the CD11b expression on B cells in PPs.

In the present study, we identified a small population of IgA^+^ B cells expressing integrin CD11b as pre-GC B cells, located outside of GCs and highly expressing *irf4*, in murine PPs. After injection of the CD11b^+^IgA^+^ PP B cells into a PP of IgA-Cre/YC3.60^flox^ reporter mice, the injected cells did not enter existing GCs directly but were located surrounding GCs, and then they entered the GCs forty-hours later, indicating that they are pre-GC IgA^+^ B cells in murine PPs. Furthermore, we found that some bacterial antigens including pam3CSK4, lipopolysaccharide (LPS) and heat-killed *Escherichia coli* (*E. coli*) or *Salmonella enterica* (*S. enterica*), but not *Bifidobacterium,* induced CD11b expression in naive B cells *in vitro*. The CD11b expression on B cells before T-B or DC-B interaction allowed the intravenously-injected B cells to enter existing GCs in PPs. Thus, B cells control their own cell fate to become pre-GC B cells, which is independent of DC activation. In addition, mice orally administered with pam3CSK4 or heat-killed *E. coli* increased the number of PP GC B cells within two days, and enhanced the mucosal antigen-specific IgA response. We propose that the induction of CD11b on activated B cells is a promising marker of pre-GC B cells as well as a useful criterion for selecting an effective mucosal vaccine adjuvant.

## Methods

### Mice

Balb/c mice (at 8–12 weeks old) were obtained from Japan CLEA. Mice were bred and maintained under specific pathogen-free conditions at the Animal Facility of the Institute for Quantitative Bioscience (IQB), the University of Tokyo. All experiments were performed following the guidelines of the Animal Care and Use Committee of IQB, the University of Tokyo.

For transfer experiments of B cells, we used IgA-Cre/YC3.60^flox^ mice (8–12 weeks-old) generously provided by Dr T. Adachi, Tokyo Medical and Dental University. Briefly, IgA-Cre mice were designed based on Allen’s paper (16). After crossing with YC3.60^flox^ mice (17, 18), IgA^+^ cells are identified as YC3.60^+^ cells.

### Flow cytometry analysis and cell sorting

PPs were carefully excised from the small intestine of Balb/c mice. Single-cell suspensions prepared from PPs were incubated with combinations of the following antibodies: Phycoerythrin-Cy7 (PE-cy7) anti-mouse/human B220 (eBioscience mAb RA3-6B2, USA), PE anti-mouse/human B220 (Biolegend mAb RA3-6B2, USA), FITC anti-mouse/human B220 (Biolegend mAb RA3-6B2, USA), PE anti-mouse IgA (alpha chain specific) (Southern Biotech, USA), Alexa Fluor (AF) 647 anti-mouse IgA (Southern Biotech, USA), FITC anti-mouse CD11b (Biolegend mAb M1/70, USA), PE anti-mouse CD11b (Biolegend mAb M1/70, USA), PE-cy7 anti-mouse CD11b (eBioscience mAb M1/70, USA), Biotinylated peanut agglutinin (PNA) (Vector Laboratories, USA), APC-R700 Hamster anti-mouse CD95 (Fas) (BD Biosciences mAb Jo2, USA), AF488 anti-mouse CD86 (Biolegend mAb GL-1, USA), APC anti-mouse CD184 (CXCR4) (Biolegend mAb L236F12, USA), PE anti-mouse IgM (eBiosciences mAb eB121-15F9, USA), AF647 anti-mouse CD4 (Biolegend mAb GK1.5, USA), streptavidin PE (Biolegend, USA), streptavidin APC (Biolegend, USA), streptavidin AF488 (Lifetechnologies, USA). An FSC-H/FSC-W gate was used to select the singlet cells. Propidium iodide (PI) (Nacalai, Japan) was used to exclude dead cells. Flow cytometry analysis was performed with a Spectral Cell Analyzer SA3800 (SONY, Japan). Cell sorting was performed with a Cell Sorter SH800 (SONY, Japan).

### Immunohistochemical analysis

Freshly isolated PPs were snap-frozen in Optimum Cutting Temperature (OCT) compound (Sakura Finetechnical, Japan) and stored at −80°C. PP sections with a thickness of 6 μm were prepared and dried overnight. On the next day, PP sections were fixed for 10 min at −20°C in acetone (Nacalai, Japan). After washing with phosphate-buffered saline (PBS) 5 times, the sections were then incubated in blocking buffer [PBS/5% FCS (NICHIREI BIOSCIENCES INC, Japan)] for 30 min. PP sections were then incubated for more than 30 min at RT in a dark box with the combination of the following antibodies: AF488 anti-mouse/human CD11b (Biolegend mAb M1/70, USA), PE anti-mouse/human CD11b (Biolegend mAb M1/70, USA), AF488 anti-mouse CD4 (Biolegend mAb GK1.5, USA), AF647 anti-mouse CD4 (Biolegend mAb GK1.5, USA), DAPI solution (BD Bioscience, USA), AF488 anti-mouse CD54 [intercellular adhesion molecule-1 (ICAM-1)] (Biolegend mAb YN1/1.7.4, USA), AF647 anti-mouse CD54 (ICAM-1 mAb YN1/1.7.4) (Biolegend, USA), Biotin anti-mouse CD11c (eBioscience mAb N418, USA), AF488 anti-mouse mucosal addressin cell adhesion molecule-1 (MAdCAM-1) (Biolegend mAb MECA-367, USA), PE anti-mouse MAdCAM-1 (Biolegend mAb MECA-367, USA), Biotinylated PNA, AF647 anti-mouse IgA (alpha chain specific) (Southern Biotech, USA), PE anti-mouse IgA (alpha chain specific) (Southern Biotech, USA), streptavidin AF488 (Lifetechnologies, USA). Sections were observed under an LSM880 microscope (Carl Zeiss, Germany). Images were analyzed with ZEN2009 software (Carl Zeiss, Germany).

### Microarray analysis

RNA was separately extracted from 25 000 sorted CD11b^+^IgA^+^ B cells and 100 000 sorted CD11b^−^IgA^+^ B cells with NucleoSpin RNA XS (Takara, Japan). Microarray analysis was performed with a SurePrint G3 Mouse GE v2 8x60K Microarray Chip (Affymetrix) to detect the gene expression.

### RNA purification and real-time PCR

Roughly 1 × 10^4^ PP GC B cells (PNA^hi^B220^+^ cells) and 1 × 10^4^ non-GC PP B cells (PNA^lo^B220^+^) were sorted, respectively. RNA was extracted from sorted cells with NucleoSpin RNA XS (Takara, Japan). cDNA was synthesized with GoScript™ Reverse Transcriptase (Promega, USA). Real-time PCR was performed in triplicate on 384-well optical PCR plates (Roche, Swiss) with KAPA SYBR^®^ FAST qPCR Master Mix (2X) Kit (KAPA Biosystems, USA) on a LightCycler 480 (Roche, Swiss). Gene expression levels were normalized with the expression level of the housekeeping gene *β-actin*. The following primers were utilized for analysis. *β-actin* fw: 5′-CCAACCGTGAAAAGAT GACC-3′, *β-actin* rv: 5′-CCAGAGGCATACAGGGACAG-3′, *itgam* (CD11b) fw: 5′-CAGATCAACAATGTGACCGTATGG G-3′, *itgam* (CD11b) rv: 5′-CATCATGTCCTTGTACTGCCGCTTG-3′, *aicda* (AID) fw: 5′-CCTACGCTACATCTCAGACT-3′, *aicda* (AID) rv: 5′-CTTTGAAGGTCATGATCCCG-3′, *bcl6* (BCL6) fw: 5′-TCCTCACGGTGCCTTTTTACA-3′, *bcl6* (BCL6) rv: 5′-TAACGACAAGCATGACGCAG-3′, *irf4* (IRF4) fw: 5′-AG GTCTGCTGAAGCCTTGGC-3′, *irf4* (IRF4) rv: 5′-CTTCAGG GCTCGTCGTGGTC-3′, *s1pr2* (S1PR2) fw: 5′-GTGACGGG ACGCAGAGGT-3′, *s1pr2* (S1PR2) rv: 5′-AAATGTCGGTGA TGTAGGCATATG-3′, *bcl2* (BCL2) fw: 5′-TGAGTACCTGAAC CGGCATCT-3′, *bcl2* (BCL2) rv: 5′-GCATCCCAGCCTCCG TTAT-3′, *gpr183* (EBI2) fw: 5′-GACATCCTGTTTACCACAGCT-3′, *gpr183* (EBI2) rv: 5′-AGACCAGAATCCAGACGGACA-3′.

### In vivo imaging

For migration imaging, IgA-Cre/YC3.60^flox^ mice (8–12 weeks-old) were anesthetized with a mixture of three types of anesthetic agents as described previously (19). An incision was carefully made in the abdominal wall, and the small intestine was exposed. PPs were identified by naked eyes. About 8000 sorted CD11b^+^IgA^+^ PP B cells and 30 000 CD11b^−^IgA^+^ PP B cells were labeled with CellTracker Orange CMTMR fluorescent dye (Invitrogen, USA) and then injected to a PP of an IgA-Cre/YC3.60^flox^ transgenic mouse directly by a 25 μl syringe (Trajan Scientific and Medical, Australia). The PP with transferred cells was observed under an LSM 880 microscope (Carl Zeiss, Germany). Images were analyzed with ZEN2009 software (Carl Zeiss, Germany). After one-hour observation of the PP under a microscope, the abdominal incision was carefully closed with an ELP Skin Stapler (Akiyama Co. Ltd. Japan). Forty hours after transfer, under anesthesia, the PP with transferred cells was observed again to identify the localization of transferred CD11b^+^IgA^+^ PP B cells under an LSM 880 microscope (Carl Zeiss, Germany) and analyzed with ZEN2009 software (Carl Zeiss, Germany).

### Conjugation analysis

Conjugations between CD11b^+^IgA^+^ PP B cells and CD4^+^ PP T cells were analyzed by flow cytometry with a Cell Sorter SH800 (SONY, Japan). Singlet cells were selected by a FSC-H/FSC-W gate. Conjugated cells were negatively selected by discrimination of the singlet cell gate. For imaging, the conjugated cells were sorted from the PP conjugated CD11b^+^IgA^+^CD4^+^ gate and observed under an LSM880 microscope (Carl Zeiss, Germany) and analyzed with ZEN2009 software (Carl Zeiss, Germany).

### Sorted CD11b^+^IgA^+^ PP B cells in the induced GC B cell culture system

About 800 sorted CD11b^+^IgA^+^ PP B cells and 800 sorted CD11b^−^IgA^+^ PP B cells were stained with a CellTrace Violet Proliferation Kit (Invitrogen, USA). Since the number of sorted CD11b^+^IgA^+^ PP B cells was very small, 5000 CD11b^−^IgA^+^ PP B cells and 5 × 10^4^ naive spleen B cells [negatively sorted by a B cell isolation kit (Miltenyi Biotec, Germany)] were prepared as positive controls. To monitor cell proliferation, the sorted CD11b^−^IgA^+^ PP B cells, CD11b^+^IgA^+^ PP B cells and naive spleen B cells were seeded in a 6-well tissue culture dish in the presence of 40LB cells that had been pre-treated with mitomycin C to inhibit the growth. Cells were cultured in RPMI-1640 medium (Wako, Japan) [containing 10% FCS, 5.5 × 10^−5^ M 2-Mercaptoethanol (ME) (Nacalai, Japan), 10 mM HEPES (Nacalai, Japan)] at 37°C with 5% CO_2_ for 3 days. Sorted CD11b^+^IgA^+^ PP B cells and CD11b^−^IgA^+^ PP B cells were cultured with rIL-21 (10 ng ml^−1^; PeproTech, USA) and naive spleen B cells were cultured with rIL-4 (1 ng ml^−1^; Biolegend, USA). Flow cytometry analysis was performed to detect CellTrace Violet from day 0 to day 3 with the iGB culture system. CD11b expression of cultured cells was analyzed on day 0 and day 1 by flow cytometry with a Cell Sorter SH800 (SONY, Japan) and a Spectral Cell Analyzer SA3800 (SONY, Japan).

### Pam3CSK4-stimulated spleen CD11b^+^ B cells in the induced GC B cell culture system

To culture spleen CD11b^+^ B cells in the induced GC B cell (iGB) culture system, spleen naive B cells were cultured with pam3CSK4 (Invivogen, USA) for 3 days to induce CD11b expression. Then, 1.5 × 10^5^ CD11b^+^ spleen B cells and 1.5 × 10^5^ CD11b^−^ spleen B cells were sorted by flow cytometry and stained with CellTrace Violet (Invitrogen, USA), and then subsequently cultured in the 40LB system with IL-4 (1 ng ml^−1^; Biolegend, USA) for 4 days. Their CD11b expression was then analyzed by flow cytometry with a Cell Sorter SH800 (SONY, Japan) and a Spectral Cell Analyzer SA3800 (SONY, Japan).

### Bacteria preparation for B cell stimulation

*E. coli* (DH5α) and *S. enterica* were inoculated from a glycerol stock into 5 ml of LB medium and then cultured by shaking at 180 rpm overnight at 37°C. On the second day, 0.5 ml of the cell suspension was re-suspended in 10 ml of LB medium. The culture was grown at 37°C with shaking (180 rpm) for 3 h. The growth curve of the culture was monitored by the optical density (OD) at the wavelength of 600 nm (OD600) detected every 30 min. One hundred μl of serial dilutions of cell suspension were spread onto the LB plate to calculate CFU. On the basis of the growth curve, we cultured and harvested *E. coli* and *S. enterica* and heat-killed them by autoclaving (121°C, 15 min).

Similarly, *Bifidobacterium bifidum* and *Bifidobacterium breve* were anaerobically cultured at 37°C in Difico Lactobacilli MRS broth (Becton, Dickinson and Company, USA) medium, and heat-killed by autoclaving.

### Spleen B cell stimulation in vitro

Spleen B cells were negatively sorted with a B cell isolation kit (Miltenyi Biotec, Germany) from spleens of 8-week-old Balb/c mice. Splenic B cells (with concentration: 5 × 10^5^ ml^−1^) were cultured in RPMI1640 medium with 13 independent stimulations, respectively. To select the proper concentration of each stimulation, we show the results of titrations of each stimulation in Supplementary Fig. 6e–g.

1. Anti-mouse IgM (15 μg ml^−1^; Jackson Immuno Research Laboratories, USA)
2. Anti-mouse-CD40 (HM40-3, 500 ng ml^−1^; Biolegend, USA)
3. B-cell activating factor (BAFF) (50 ng ml^−1^; Biolegend, USA)
4. Pam3CSK4 (1 μg ml^−1^, Invivogen, USA)
5. Poly I:C (25 ng ml^−1^, Invivogen, USA)
6. LPS (50 ng ml^−1^; Sigma-Aldrich, USA)
7. Flagellin (20 ng ml^−1^; Sigma-Aldrich, USA)
8. Imiquimod (R837, 5 ng ml^−1^; Invivogen, USA)
9. CpG (20 μg ml^−1^, GeneDesign Inc, Japan)
10. Heat-killed *E. coli* (10^7^ CFU ml^−1^)
11. Heat-killed *S. enterica* (10^8^ CFU ml^−1^)
12. Heat-killed *B. bifidum* (10^7^ CFU ml^−1^)
13. Heat-killed *B. breve* (10^7^ CFU ml^−1^)

CD11b expression of stimulated B cells was analyzed on day 0 and day 3 with a Cell Sorter SH800 (SONY, Japan) and a Spectral Cell Analyzer SA3800 (SONY, Japan).

For muramyl dipeptide (MDP) stimulation, purified splenic B cells (5 × 10^5^ ml^−1^) were cultured in RPMI1640 medium with N-acetylmuramyl-L-alanyl-D-isoglutamine hydrate (1 μg ml^−1^; Sigma-Aldrich, USA).

For toll-like receptor (TLR) inhibitor and NOD2 inhibitor analysis, purified splenic B cells (5 × 10^5^ ml^−1^) were cultured in RPMI1640 medium with the TLR1/2 antagonist, CU-CPT (1 μM; Sigma-Aldrich, USA), TLR4 inhibitor, TAK-242 (1 μM; Sigma-Aldrich, USA) or NOD2 signaling inhibitor II, GSK717 (30 μM; Sigma-Aldrich, USA) in the presence of pam3CSK4 (1 μg ml^−1^, Invivogen, USA), LPS (50 ng ml^−1^; Sigma-Aldrich, USA), CpG (20 μg ml^−1^, GeneDsign Inc, Japan) and heat-killed *E. coli* (10^7^ CFU ml^−1^).

### Immunization

On days 0 and 16, mice were orally administered with ovalbumin (OVA) (1 mg), OVA (1 mg) + pam3CSK4 (10 μg, Invivogen, USA) or OVA (1 mg) + heat-killed *E. coli* (10^9^ CFU in 100 μl). On day 21, feces were collected for OVA-specific antibody measurement by enzyme-linked immunosorbent assay (ELISA).

### Detection of OVA-specific IgA ELISA

Feces were suspended in 10 times weight/volume (w/v) of PBS. After centrifugation (8000 × *g* for 15 min), supernatants were collected as fecal extracts. Plates were pre-coated with 1 mg ml^−1^ OVA overnight, followed by blocking for 1 h at room temperature with PBS containing 1% (w/v) bovine serum albumin. Then fecal extracts with serial dilutions were added for incubation for 1 h at room temperature. The relative binding ability of IgA was detected with alkaline phosphatase-conjugated goat anti-mouse IgA (Southern Biotech, USA). After incubation at 4°C overnight, the OD values at 405 nm were measured with a TriStar Multimode Reader LB 942 (BERTHOLD, Germany).

### Oral administration with heat-killed bacteria to WT mice

Balb/c mice (at 8–12 weeks old) were orally administered with heat-killed *E. coli* (10^9^ CFU diluted by 100 μl PBS per mouse), *S. enterica* (10^9^ CFU diluted by 100 μl PBS per mouse), *B. bifidum* (10^9^ CFU diluted by 100 μl PBS per mouse), *B. breve* (10^9^ CFU diluted by 100 μl PBS per mouse) and pam3CSK4 (10 μg diluted by 100 μl PBS per mouse). Mice orally administered with 100 μl PBS were prepared as controls. Two days later, PPs from those mice were prepared to analyze their GC B cells with a Spectral Cell Analyzer SA3800 (SONY, Japan), as described above.

### In vivo imaging of transferred iGB cells induced by pre-stimulation

As described above, spleen naive B cells were cultured with pam3CSK4 (1 μg ml^−1^, Invivogen, USA), heat-killed *E. coli* (10^7^ CFU ml^−1^), CpG (20 μg ml^−1^, GeneDsign Inc, Japan) or anti-IgM-Ab (15 μg ml^−1^; Jackson Immuno Research Laboratories, USA) for 3 days *in vitro*, separately. Then, 1 × 10^5^ B220^+^ B cells were sorted from the simulated cells and subsequently seeded on 40LB feeder cells with rIL-4 (1 ng ml^−1^; Biolegend, USA) to induce iGB cells. Non-stimulated spleen naive B cells were seeded on 40LB feeder cells with rIL-4 (1 ng ml^−1^; Biolegend, USA) as a control group.

After 4-day culturing, 3 × 10^5^ iGB cells (B220^+^) of naive B-40LB cells, pam3CSK4-40LB cells, *E. coli*-40LB, IgM-40LB and CpG-40LB cells were sorted and labeled with CellTracker Orange CMTMR fluorescent dye (Invitrogen, USA) and then separately injected intravenously to a mouse (Balb/c, 8–12 weeks old) together with 10 μg of AF488 anti-mouse MAdCAM-1 (Biolegend mAb MECA-367, USA) to identify high endothelial venules (HEVs). Under anesthesia, to stain the IgA^+^ cells for GC identification, 1 μg of AF647 anti-mouse IgA (Southern Biotech, USA) was directly injected to a PP of the same mouse transferred with iGB cells. The localization of transferred labeled iGB cells was observed under an LSM880 microscope (Carl Zeiss, Germany) and analyzed with ZEN2009 software (Carl Zeiss, Germany).

### DNA amplification of the rearranged VDJ region and downstream intron

Rearranged VDJ sequences with downstream intronic sequences were amplified by two rounds of nested PCR with several different upstream primers (where ‘S’ is C or G; ‘R’ is A or G; ‘N’ is A, G, C or T; ‘M’ is A or C; and ‘W’ is A or T): MH1, 5′-SARGTNMAGCTGSAGSAGTC-3′; MH2, 5′-SARGTNMAGCTGSAGSAGTCWGG-3′; MH3, 5′-CAGGTTACTCTGA AAGWGTSTG-3′; MH4, 5′-GAGGTCCARCTGCAACARTC-3′; MH5, 5′-CAGGTCCAACTVCAGCARCC-3′; MH6, 5′-GAGG TGAASSTGGTGGAATC-3′; MH7, 5′-GATGTGAACTTGGAAG TGTC-3′, together with a JH4R primer that is complementary to a sequence located at the 5′-end of the IgH intronic enhancer (5′-GACTAGTCCTCTCCAGTTTCGGCTGAATCC-3′) (20, 21). The second round of PCR was carried out with the same upstream primers together with a JH4R-2 primer (5′-CAGGTGGTGTTTTGCTCA-3′) that is complementary to an upstream sequence of the JH4R primer. Amplification was carried out with 20 cycles of first round PCR and 30 cycles of second round PCR (95°C 15 s, 58°C 10 s, 72°C 30 s) with PrimeSTAR Max DNA Polymerase (TaKaRa, Japan). After cloning the PCR products into pGEM T vector (Promega, USA), clones containing the intronic sequence were selected by colony PCR with the JH4F primer (5′-TAT GCT ATG GAC TAC TGG-3′) and JH4R-2 primer with EmeraldAmp® PCR Master Mix (TAKARA, Japan).

### DNA sequencing and SHM analysis

After plasmid DNA isolation, DNA sequence analysis was performed with T7 and SP6 primers. The consensus of the IgH intronic DNA sequence was retrieved from the Accession No. AJ 851868.3. The rearranged VDJ was assigned with the IgBLAST.

### Statistical analysis

Except when otherwise stated, statistical analyses were performed using GraphPad Prism version 8.4.2 for Mac (GraphPad Software, San Diego, CA, USA). Differences between two individual groups were compared using a two-tailed unpaired Student’s *t* test. In the case of more than three groups, one-way or two-way analysis of variance (ANOVA) followed by Tukey’s multiple comparisons test was performed. The statistical test and details about group number and replicates are indicated in the figure legends. A *P*-value < 0.05 was considered significant.

## Results

### A small population of IgA^+^ B cells expressing CD11b in murine PPs

**Fig. 1.**
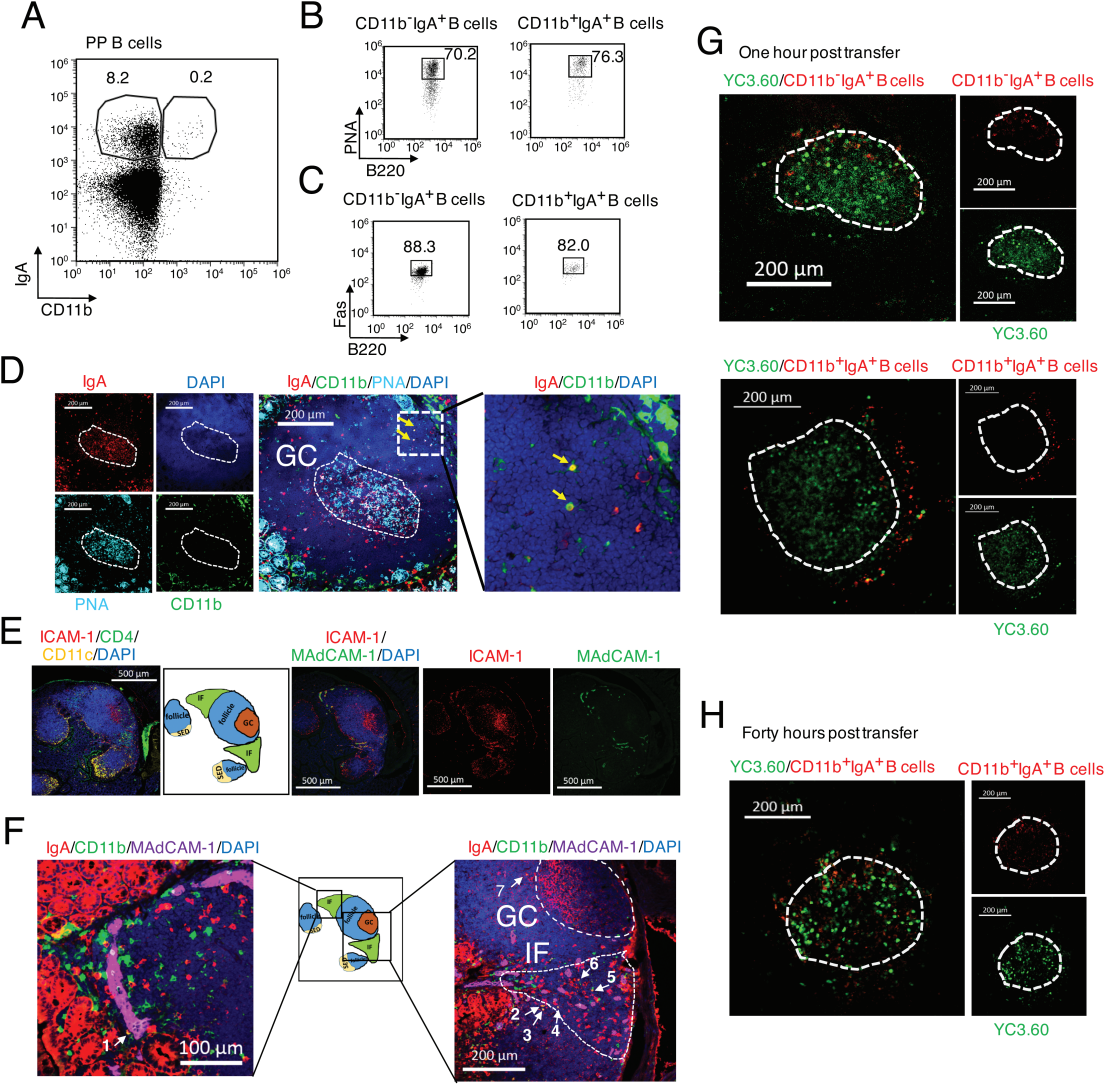
The CD11b^+^IgA^+^ PP B cells are located outside GCs migrating towards GCs. **(A)** Representative flow cytometry data of IgA and CD11b expression in the PP B220^+^ B cells from WT mice. **(B, C)** Flow cytometry of GC B cells in CD11b^+^IgA^+^ and CD11b^−^IgA^+^ PP B cells, stained with B220 and PNA **(B)** or B220 and Fas **(C**). **(A–C)** Numbers indicate the percentage of cells. Representative data are from at least 20 experiments. **(D)** Microscopic images of PP section stained with PNA (cyan), CD11b (green), IgA (red) and DAPI (blue). Two CD11b^+^IgA^+^ double positive cells (yellow arrows) were found outside the GC (dashed circle). Scale bar, 200 μm. **(E)** Images show the SED, IF, GC areas stained with ICAM-1 (red), CD11c (yellow), CD4 (green, in the left photo), MAdCAM-1 (green, in right photos) and DAPI. Each area is marked in the middle schema. Scale bar, 500 μm. **(F)** Images show that CD11b^+^IgA^+^ B cells (white arrows, marked as 1–7) are located in the indicated areas. The GC and IF areas are marked by dashed lines. Scale bar, 200 μm. **(G)** CD11b^−^IgA^+^ B cells and CD11b^+^IgA^+^ B cell were directly injected into a PP of IgA-Cre/YC3.60^flox^ mice, respectively. One hour after injection, representative images show the location of transferred CD11b^+^IgA^+^ PP B cells (red, lower images) or CD11b^−^IgA^+^ PP B cells (red, upper images) in the PP of IgA-Cre/YC3.60^flox^ mice. The GC boundary (dashed white line) was identified as an IgA-YC3.60 (green) enriched area. Scale bar, 200 μm. **(H)** Forty hours after injection, the PP with injected CD11b^+^IgA^+^ B cells (red) was observed by microscopy. Scale bar, 200 μm. Data are representative from three independent experiments.

By flow cytometry, we identified a small population of CD11b^+^IgA^+^ B cells in PPs (about 0.1%–0.2% of PP B cells) (Fig. 1A, Supplementary Fig. 1A). Both the CD11b^+^IgA^+^ and CD11b^−^IgA^+^ PP B cells showed typical GC B cell phenotypes, such as PNA^high^ and Fas^+^ (21) (Fig. 1B and C).

Next, we investigated the localization of CD11b^+^IgA^+^ B cells, since pre-GC B cells are located in the IF area (outside GCs) before entering GCs (3, 4). By immunohistology, the GC area was identified by both PNA and IgA expression (Fig. 1D). We found that only two CD11b^+^IgA^+^ B cells (yellow cells) reside in the area outside the GC in the section (Fig. 1D, Supplementary Fig. 2), which is a reasonable result because the number of CD11b^+^IgA^+^ B cells is very small (Fig. 1A). We subsequently analyzed the localization of the CD11b ligand. ICAM-1 is widely known as the ligand of CD11b/CD18 that mediates cell adhesion and migration (22). As reported previously (8, 23), both GC B cells and HEVs in IF area express ICAM-1. The HEVs also express MAdCAM-1 (24) (Fig. 1E, Supplementary Fig. 3). Although we did not find the CD11b^+^IgA^+^ PP B cells directly binding to HEVs, we found that 6 out of 7 CD11b^+^IgA^+^ PP B cells were located near HEVs in the IF area in a section of PPs (Fig. 1F). Another CD11b^+^IgA^+^ PP B cell (No. 7) was located near the GC (Fig. 1F), but not inside the GC, suggesting that it may be migrating from the IF to GC.

The next question is whether CD11b^+^IgA^+^ PP B cells subsequently migrate into the GCs from outside of GCs. We sorted CD11b^+^IgA^+^ and CD11b^−^IgA^+^ PP B cells, and then labeled and separately injected them into a PP from a different IgA-Cre/YC3.60^flox^ reporter mouse. Since PP GCs can be identified by the IgA^+^ B cell-enriched area (Fig. 1D), these reporter mice with YC3.60 fluorescence at IgA^+^ cells enable us to readily identify PP GCs. One hour after injection, the injected CD11b^−^IgA^+^ B cells were located inside the GCs (Fig. 1G), suggesting that CD11b^−^IgA^+^ B cells are GC B cells. In contrast, the injected CD11b^+^IgA^+^ B cells did not enter GCs but surrounded them (Fig. 1G). Forty hours after injection, the injected CD11b^+^IgA^+^ B cells also entered the GC (Fig. 1H). Thus, CD11b^+^IgA^+^ B cells originally located in the IF area will migrate into GCs.

### Gene expression of CD11b^+^IgA^+^ PP B cells

**Fig. 2.**
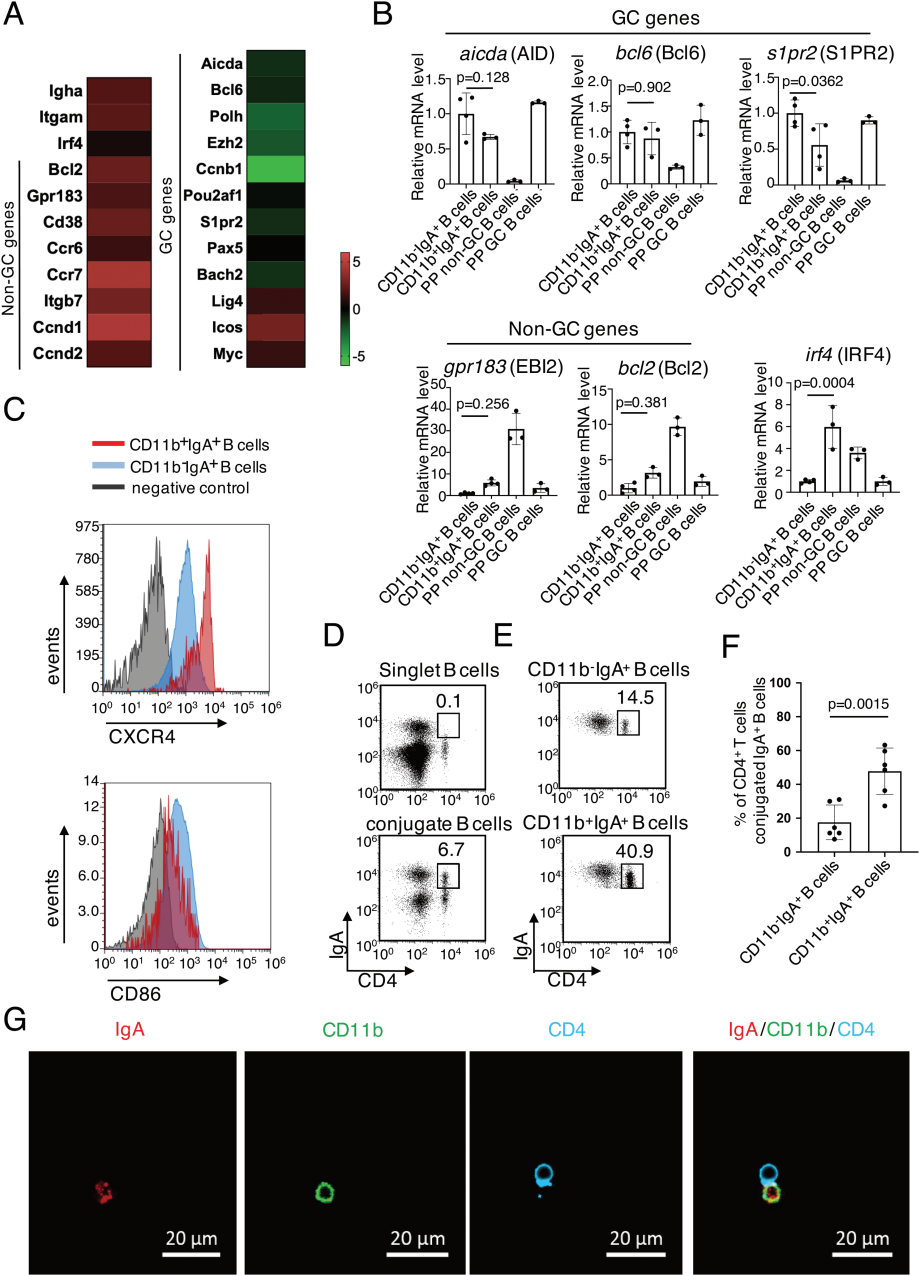
CD11b^+^IgA^+^ B cells are pre-GC B cells interacting with CD4^+^ T cells. **(A)** Heatmap indicating the log fold change (CD11b^+^IgA^+^ B cells/CD11b^−^IgA^+^ B cells) in expression of indicated genes by microarray analysis. **(B)** Expression levels of indicated GC specific genes and non-GC specific genes and a pre-GC gene were analyzed by qPCR and normalized to those from the *β-actin* gene. The mean of relative expression levels of CD11b^−^IgA^+^ B cells is taken as 1. Each spot represents the mean of technical triplicates. Bar graphs show the mean values (±SD) of three or four independent measurements. Data were analyzed by ANOVA followed by Tukey’s multiple comparisons. **(C)** Flow cytometry histograms depicting expression of surface markers of CXCR4 and CD86 in CD11b^+^IgA^+^ B cells and CD11b^−^IgA^+^ B cells. Representative data are from at least five independent experiments. **(D)** Flow cytometry data of singlet IgA^+^ B cells and conjugated CD4^+^ IgA^+^ cells. **(E)** Flow cytometry data of CD11b^−^IgA^+^ and CD11b^+^IgA^+^ PP B cells with CD4^+^ T cells in the conjugated gate. **(F)** Percentage of the CD4^+^ T cells in conjugation with CD11b^+^IgA^+^ and CD11b^−^IgA^+^ PP B cells. Bar graphs show the mean values (±SD) of six independent measurements. Data were compared by two-tailed unpaired Student’s t test. **(G)** Imaging of sorted CD11b^+^IgA^+^ PP B cells conjugating with CD4^+^ T cells. Scale bar, 20 μm.

To further confirm the pre-GC B cell phenotypes, we sorted the CD11b^+^IgA^+^ and CD11b^−^IgA^+^ PP B cells and compared their gene expression by microarray analysis (Fig. 2A and Supplementary Fig. 1B). We confirmed the gene expressions of *itgam* (CD11b) and *igha* (IgA) in CD11b^+^IgA^+^ PP B cells (Fig. 2A). CD11b^+^IgA^+^ B cells expressed GC genes at relatively lower levels and non-GC genes at relatively higher levels than CD11b^−^IgA^+^ B cells, indicating that CD11b^+^IgA^+^ B cells are a distinct population from CD11b^−^IgA^+^ B cells. To confirm our microarray result, we further performed quantitative PCR (qPCR) (Fig. 2B and Supplementary Fig. 1B) to compare the gene expression of CD11b^+^IgA^+^ B cells with that of sorted GC (PNA^high^B220^+^) and non-GC (PNA^low^B220^+^) PP B cells. We selected three genes highly expressed in GC B cells, including *bcl6* (a main transcription factor restricted in GC B cells), *aicda* [encoding activation-induced cytidine deaminase (AID), an enzyme inducing both SHM and class-switch recombination] and *s1pr2* (a receptor regulating B cell position outside and inside GCs) (10, 25–27) and two non-GC genes, the *gpr183* (a receptor regulating B cell position outside the follicle) and *bcl2* (a transcription factor highly expressed in non-GC B cells) (10, 28, 29). The expression levels of these genes of both CD11b^+^IgA^+^ and CD11b^−^IgA^+^ PP B cells are distinct from those of the non-GC B cells (Fig. 2B), indicating both the two populations are likely GC B cells. Since pre-GC B cells up-regulate *irf4*, a transcription factor that has been demonstrated to be necessary for the initiation of the GC reaction (10, 11, 30), we further analyzed the expression of *irf4*. CD11b^+^IgA^+^ PP B cells express higher *irf4* than CD11b^−^IgA^+^ PP B cells (Fig. 2B), indicating that they are pre-GC B cells. However, considering that some GC LZ B cells also highly express *irf4* (8, 30), we subsequently analyzed the CXCR4 and CD86 expression of CD11b^+^IgA^+^ PP B cells by flow cytometry. Since GC LZ B cells are identified as CD86^high^CXCR4^low^ B cells (3, 8), the lower expression of CD86 and higher expression of CXCR4 in CD11b^+^IgA^+^ B cells compared with CD11b^−^IgA^+^ B cells (Fig. 2C, Supplementary Fig. 1C) demonstrated that CD11b^+^IgA^+^ B cells are not GC LZ B cells, but are pre-GC B cells.

### CD11b^+^IgA^+^ PP B cells interact with T cells before entering GCs

To enter GCs, the pre-GC B cells must interact with T cells or DCs, as reported previously (3, 31). So, we checked whether CD11b^+^IgA^+^ PP B cells interact with CD4^+^ T cells before entering GCs. IgA^+^ PP B cells were not CD4^+^ in the singlet B cell gate by flow cytometry (Fig. 2D, Supplementary Fig. 4). However, a distinct population of IgA^+^CD4^+^ double positive PP cells exist in the conjugated B cells (Fig. 2D, Supplementary Fig. 4), indicating that these IgA^+^ PP B cells are interacting with CD4^+^ T cells. In particular, more than 40% of CD11b^+^IgA^+^ B cells showed CD4^+^ T cell-conjugation (Fig. 2E and F, Supplementary Fig. 4), suggesting that CD11b^+^IgA^+^ PP B cells are interacting with CD4^+^ T cells. On the other hand, about 14.5% of CD11b^−^IgA^+^ PP B cells are interacting with CD4^+^ T cells, maybe CD4^+^ T_FH_ cells in the GC LZ (Fig. 2E and F) (4, 8). Confocal microscopic imaging of the conjugated cells sorted from PPs further confirmed the interaction between CD11b^+^IgA^+^ PP B cells and CD4^+^ T cells (Fig. 2G). Thus, our results clearly indicate that CD11b^+^IgA^+^ PP B cells are pre-GC PP B cells.

### CD11b^+^IgA^+^ PP B cells accumulate SHM in their Ig genes

To check whether CD11b^+^IgA^+^ B cells are derived from newly activated naive B cells, we sequenced both the rearranged VDJ region and the downstream intronic sequences of IgH of CD11b^+^IgA^+^ and CD11b^−^IgA^+^ PP B cells, where SHM is introduced during the GC reaction (Supplementary Fig. 5A and B) (21). More than 80% of the sequences from CD11b^+^IgA^+^ PP B cells (21 out of 25 clones), as well as CD11b^−^IgA^+^ PP B cells (32 out of 39 clones), have mutations (Supplementary Fig. 5B), indicating that CD11b^+^IgA^+^ PP B cells are derived not only from newly activated naive B cells but also from memory B cells re-entering the GC, as reported previously (6, 7).

### Bacterial antigens induce CD11b expression on naive spleen B cells

**Fig. 3.**
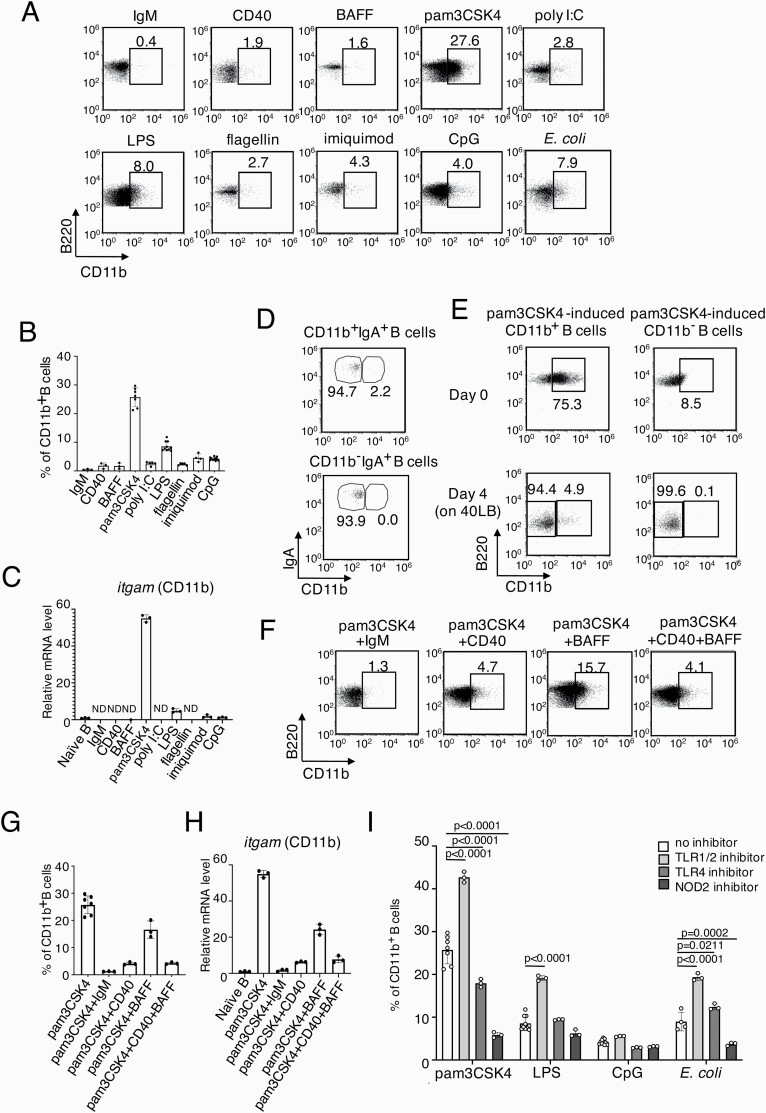
Bacterial antigens induce CD11b expression on B cells. **(A, F)** Flow cytometry data of the CD11b expression of the spleen naive B cells stimulated with indicated stimulations for 3 days. **(B, G)** Percentages of CD11b^+^ B cells of each stimulation. **(C, H)** qPCR analysis of *itgam* with the sorted B cells after indicated stimulations. The mean of relative expression levels of spleen naive B cells was taken as 1. **(D)** Flow cytometry data of sorted CD11b^+^IgA^+^ and CD11b^−^IgA^+^ PP B cells after one day culture on 40LB cells. **(E)** Pam3CSK4-induced CD11b expression was lost by culture on 40LB cells. **(I)** Percentages of CD11b^+^ B cells of each stimulation with indicated inhibitors. **(B, C, G, H, I)**, Bar graphs show the mean values (±SD) of at least three independent measurements. **(B, C, G, H)** Data were analyzed by one-way ANOVA followed by Tukey’s multiple comparisons. **(I)** Data were analyzed by two-way ANOVA followed by Tukey’s multiple comparisons.

Our findings concerning CD11b^+^IgA^+^ PP B cells raise another question of what triggers CD11b expression on pre-GC B cells. Besides stimulating antigens, B cells also interact with DCs and T cells to be transformed into pre-GC B cells (3, 4, 31). To investigate which signal induces CD11b expression, we sorted the naive spleen B cells from unimmunized mice and cultured them *in vitro* with anti-IgM for BCR crosslinking, BAFF for interaction with DCs and anti-CD40 for interaction with T cells. However, these stimulations mimicking T-B and DC-B interactions did not induce CD11b on the naive B cells. Next, we stimulated the naive B cells with different TLR ligands, including pam3CSK4 for TLR2, poly I:C for TLR3, LPS for TLR4, flagellin for TLR5, imiquimod for TLR7 and CpG for TLR9. After culturing for 3 days, the number of B cells stimulated with pam3CSK4, LPS and CpG significantly increased to levels more than those stimulated with anti-IgM, anti-CD40, BAFF, poly I:C, flagellin or imiquimod (Supplementary Fig. 6A and B). The percentages of the CD11b^+^ B cells in the B cells stimulated with pam3CSK4 and LPS were 27.6% and 8%, respectively, while those in B cells stimulated with the other stimulations were less than 5% (Fig. 3A and B). It is notable that CpG did not induce CD11b expression, although it activated B cell proliferation. We further confirmed that *itgam* (CD11b) expression in the B cells increased only when stimulated with pam3CSK4 or LPS that are known as bacterial antigens (Fig. 3C). We then prepared heat-killed *E. coli,* which is a strong stimulant for both TLR2 and (or) TLR4 (32, 33)_,_ and found that heat-killed *E. coli* significantly induced CD11b expression on spleen B cells (Fig. 3A). These results showed that the bacterial antigens, TLR2 and TLR4 ligands, induced CD11b on B cells.

Since the B cells inside PP GCs do not express CD11b (Fig. 1D), we hypothesized that CD11b expression on B cells may be transient and convertible via T-B or DC-B interaction. To follow the surface phenotypes, we utilized an *in vitro* iGB culture system that mimics the role of T cells and DCs for inducing GC-like B cells on the feeder layer of 40LB cells expressing CD40L and BAFF (31). After the cell culture with the iGB system, the sorted CD11b^+^IgA^+^ PP B cells can proliferate at similar levels as the sorted CD11b^−^IgA^+^ PP B cells or naive spleen B cells (Supplementary Fig. 7A and B). Indeed, the sorted CD11b^+^IgA^+^ PP B cells lost their CD11b expression after one day of culture with the iGB system (Fig. 3D, Supplementary Fig. 7A). Similarly, the pam3CSK4-induced CD11b^+^ spleen B cells lost the CD11b expression after culturing with the iGB system for 4 days (Fig. 3E, Supplementary Fig. 7C). Spleen naive B cells treated with anti-CD40 Ab and anti-IgM Ab significantly suppressed CD11b expression, even in the presence of pam3CSK4 (Fig. 3F–H), indicating that T-B cell interaction and BCR-crosslinking down-regulate the CD11b expression on B cells. Therefore, it is reasonable that we found quite a few CD11b^+^IgA^+^ PP B cells *in vivo* (Fig. 1).

To further confirm whether TLR2 and TLR4 play a role in the induction of CD11b expression of spleen B cells, we stimulated spleen B cells with pam3CSK4, LPS, CpG, and heat-killed *E. coli* in the presence of a TLR1/2 inhibitor, TLR4 inhibitor or NOD2 inhibitor. NOD2 is known to mediate in the downstream signaling of TLR2 (34). Interestingly, the TLR1/2 inhibitor, pam3CSK4 and heat-killed *E. coli* increased the percentages of CD11b^+^ B cells, while the TLR4 inhibitor did not (Fig. 3I). On the other hand, the NOD2 inhibitor strongly suppressed CD11b expression on B cells stimulated with pam3CSK4 and heat-killed *E. coli,* although muramyl dipeptide (MDP), the ligand of NOD2, did not induce CD11b expression (Supplementary Fig. 6D). This observation strongly suggests that CD11b expression is not directly induced by TLR2 or TLR4 activation, but partly by NOD2-mediated stimuli derived from bacteria (Fig. 3I).

### Oral administration with heat-killed CD11b-inducible bacteria enhances the GC reaction

**Fig. 4.**
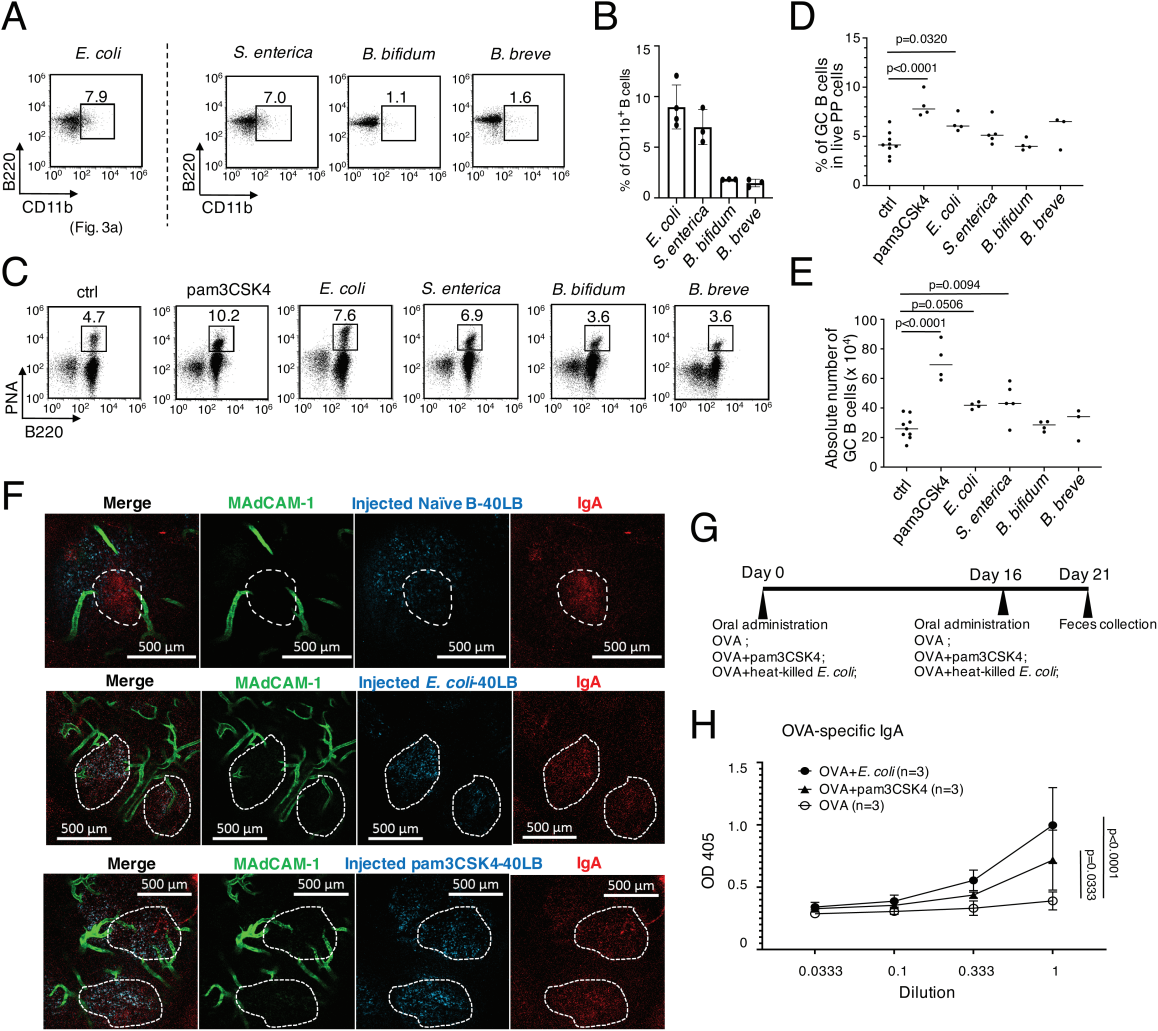
CD11b inducible bacterial antigens enhance the existing GC reaction in PPs. **(A)** Flow cytometry data of the CD11b expression of the spleen naive B cells stimulated with indicated stimulations for three days. **(B)** Percentages of CD11b^+^ B cells for each stimulation. **(C–E)** Balb/c mice were orally administered with indicated heat-killed bacteria, pam3CSK4 or PBS (ctrl). Two days later, flow cytometry data **(C)** of PNA^hi^B220^+^ GC B cells, percentages **(D)** and absolute numbers (**E**) of PNA^high^ B220^+^ PP GC B cells from each mouse were calculated. **(F)** Indicated iGB cells were sorted, labeled (cyan) and then intravenously injected to mice, independently. One of the PPs was selected to analyze the injected cell localization. To identify the GC boundary (dashed white line), anti-IgA was directly injected into the selected PP. Scale bar, 500 μm. **(G)** Schedule for oral immunization. **(H)** OVA-specific IgA after immunization. **(B, D, E, H)** Bar graphs show the mean values (±SD) of at least three independent measurements. **(D, E)** Data were analyzed by one-way ANOVA followed by Tukey’s multiple comparisons. **(H)** Data were analyzed by two-way ANOVA followed by Tukey’s multiple comparisons.

The next question is what kind of bacteria can induce pre-GC B cells through CD11b expression. We selected *E. coli* and *S. enterica* as harmful bacteria, and *B. bifidum* and *B. breve* as representative beneficial bacteria. By *in vitro* stimulation, *E. coli* and *S. enterica* induced expression of CD11b, while *B. bifidum* and *B. breve* did not (Fig. 4A and B). After oral administration of pam3CSK4 or heat-killed bacteria to mice, the numbers and percentages of PP GC B cells increased in mice administered with pam3CSK4, *E. coli* and *S. enterica* in two days (Fig. 4C–E), suggesting that only harmful bacteria are capable of enhancing the ongoing GC reaction *in vivo*.

### CD11b transient expression enables cultured B cells to enter GCs

Next, we checked whether *in vitro*-induced CD11b^+^ B cells can enter the existing GCs. To induce GC B cells *in vitro*, we stimulated the spleen naive B cells with pam3CSK4 or heat-killed *E. coli in vitro* to induce CD11b, and subsequently cultured them on 40LB cells for 4 days. Notably, most of the iGB cells pre-stimulated with pam3CSK4 or heat-killed *E. coli,* CD11b-inducible stimuli, entered GCs after intravenous injection to mice, while iGB cells without CD11b expression, pre-stimulated with bacterial antigen, such as CpG and anti-IgM, did not enter GCs (Fig. 4F, Supplementary Fig. 8). Taken together, our results show that CD11b expression on B cells before T-B or DC-B interaction is essential for pre-GC B cells to enter GCs, which is consistent with a previous report that bacterial antigens, as non-self antigens, are important to evoke the GC reaction (35).

### CD11b-inducible heat-killed bacteria function as an effective mucosal adjuvant and induce antigen-specific mucosal IgA

We note that TLR2 or TLR9 ligand-based adjuvants are already widely used for vaccination, since they stimulate DCs efficiently (36, 37). However, our results demonstrate that B cells can determine their own cell fate to pre-GC B cells before receiving help from DC and T cells. Therefore, we hypothesized that pam3CSK4 and heat-killed *E. coli* can work as mucosal vaccine adjuvants. We orally administered mice with OVA as an antigen plus pam3CSK4 or heat-killed *E. coli* as adjuvants (Fig. 4G). As we expected, mice administered simultaneously with OVA plus heat-killed *E. coli* or pam3CSK4 increased OVA-specific IgA in their feces, but those administered with OVA only did not (Fig. 4H). Thus, our study revealed that the pre-GC surface marker, CD11b, on B cells is a promising marker for selecting an effective mucosal vaccine adjuvant, especially to enhance GC-derived high-affinity mucosal IgA.

## Discussion

In this study, by investigation of the distinct population of CD11b^+^IgA^+^ PP B cells, we show that CD11b is a novel surface marker of pre-GC IgA^+^ B cells in murine PPs. CD11b induction on B cells is dependent on harmful bacterial antigen stimulation but independent of activated DCs. Mice orally administered with those CD11b-inducing bacterial antigens showed enhanced mucosal antigen-specific IgA response *in vivo*. Our results demonstrate that transient CD11b expression on activated B cells before entering GC is an important step to select activated B cells appropriate for the GC response.

What is the function of CD11b on B cells? Yan’ s group has shown that CD11b is critical in regulating BCR signaling via the Lyn-CD22-SHP-1 negative feedback pathway (13). CD11b^−/−^ mice exhibited enhanced antibody production and GC responses with autoreactive B cells (13, 14). Accordingly, the mutations in the *ITGAM* gene (encoding CD11b) are reported as a high-risk factor of developing autoimmune diseases, such as systemic lupus erythematosus (SLE) (38). In our study, we identified CD11b expression as a pre-GC B cell marker for entering GCs. Probably, CD11b transient expression is sufficient, but not necessary, for entering GCs. Once B cells are activated by bacterial stimuli, they express CD11b to avoid their hyperproliferation and production of potential autoreactive antibodies. Subsequently, B cells begin to interact with T cells in the IF area. After T-B interaction, activated B cells lose their CD11b expression, enter the GC DZ and proliferate fast and undergo SHM for obtaining high affinities. We propose that CD11b plays a key role in controlling the activated B cells for undergoing beneficial GC reactions but not for harmful autoreactive responses. The precise selection mechanism requires further investigation.

Do human B cells also express CD11b induced by microbial antigens? Dutra’s group has demonstrated that stimulation with a *Trypanosoma cruzi*-derived protein-enriched fraction leads to a higher frequency of CD11b^+^ B cells after stimulation of B cells from the patients with Chagas disease (39). However, whether the CD11b expression in humans is associated with the GC reaction remains unknown.

We also addressed the question of which signal induces CD11b expression. At first, we expected that CD11b is induced by TLR2 stimulation, since pam3CSK4, a TLR2 ligand, and heat-killed *E. coli*, strongly induced CD11b on B cells *in vitro*. *E. coli* have peptidoglycan (PGN) as a component of the bacterial cell wall, a TLR2 ligand (40). However, unexpectedly pam3CSK4 plus a TLR1/2 inhibitor enhanced CD11b expression on B cells. On the other hand, CD11b expression induced by pam3CSK4 and heat-killed *E. coli* was suppressed by a NOD2 inhibitor (Fig. 3I), suggesting NOD2-mediated CD11b regulation. Our results partly agree with a previous study that NOD2 can work as a negative regulator of TLR2 (40). In addition, NOD2 helped to mediate immune tolerance and homeostasis by inhibiting TLR2/4-mediated induction of inflammatory cytokine production, which is strongly associated with Crohn’s disease (CD) (41). Thus, our data suggest one possibility that the TLR2-NOD2 pathway may regulate not only the inflammatory response but also the GC reaction through CD11b regulation. Other studies, however, have demonstrated that the TLR1/2 signaling pathway is independent from NOD2 (42). Taken together with enhanced CD11b induction by the TLR2 inhibitor, there is controversy to be solved.

In terms of CD11b-inducible bacterial stimuli, we have shown that *S. enterica* and *E. coli* are examples of harmful bacteria that induce CD11b expression *in vitro* and enhance the GC reaction *in vivo*, while *Bifidobacterium* species do not. Whether CD11b induction can be distinguished by the components of beneficial bacteria from those of harmful bacteria is still not fully investigated. Further study is necessary to answer the question of what are the exact stimuli for CD11b expression.

## Supplementary Material

dxab113_suppl_Supplementary_Figures

## Data Availability

All data are available in the main text or the supplementary materials.
