# Supplementary material for: Integrin CD11b provides a new marker of pre-germinal center IgA+ B cells in murine Peyer’s patches
Source: Int Immunol. 2021 Dec 31;34(5):249–62. doi: 10.1093/intimm/dxab113 (PMC9020567; doi:10.1093/intimm/dxab113)
Supplement: dxab113_suppl_Supplementary_Figures [file dxab113_suppl_supplementary_figures.pdf]

Figure S1

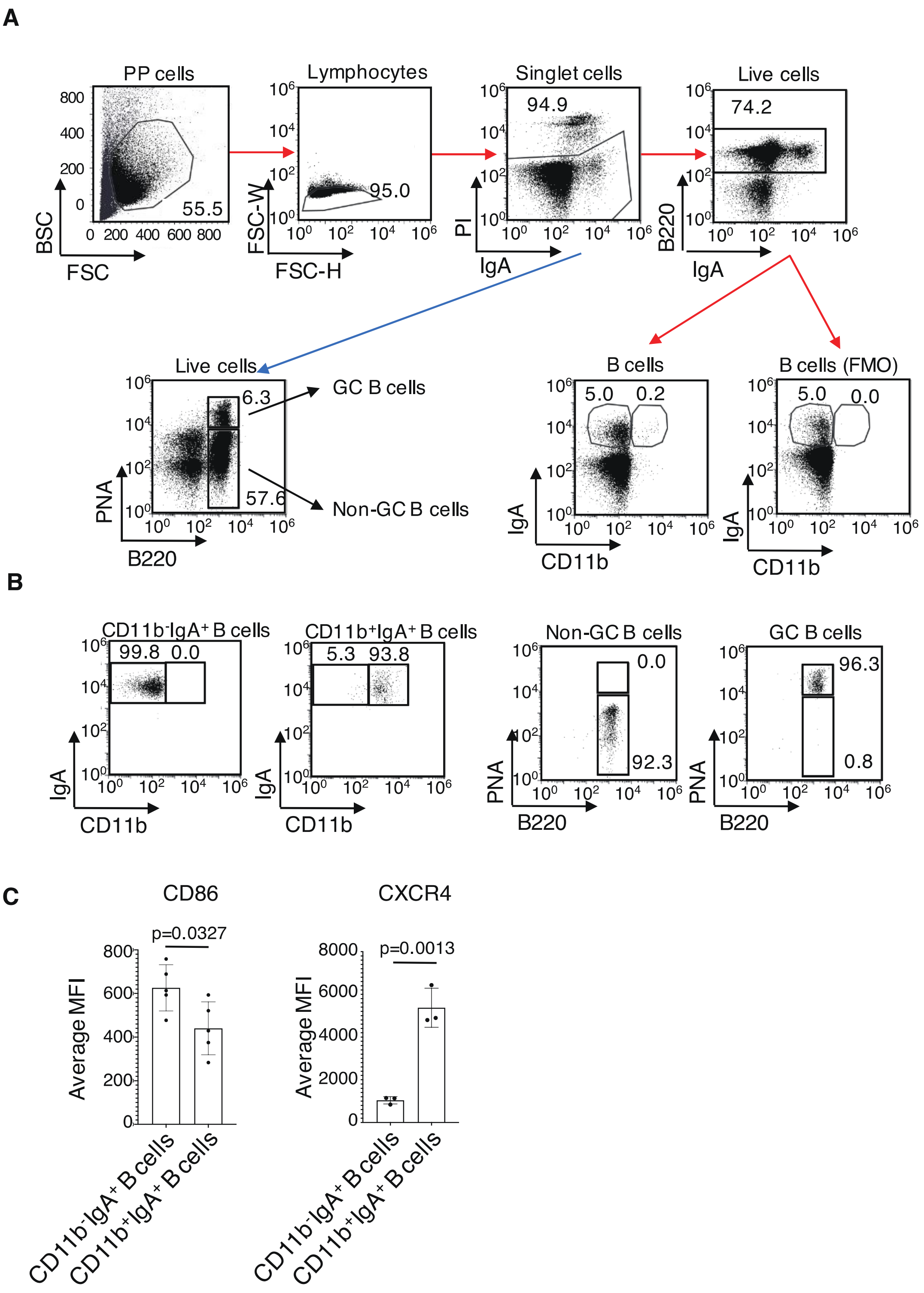

Extended Data Fig. 1. CD11b^{+}IgA^{+} B cells exist in murine PPs.

(A) Flow cytometry analysis was performed with WT Balb/c mice of 8-12-week-old. Doublet cells were discriminated by FSC-H/FSC-W gate. Dead cells were excluded by propidium iodide (PI) staining. IgA and CD11b expressions were analyzed in the $B220^{+}$ B cells. To confirm the CD11b expression, fluorescence minus one (FMO) sample was prepared as negative control. In live cells, GC B cells represent $PNA^{high}B220^{+}$ cells, non-GC B cells represent $PNA^{low}B220^{+}$ cells. Representative data are from at least 20 experiments. (**B**) The sorting purity was confirmed at least 90%. All data are from at least three independent experiments. **(C)** Average mean fluorescence intensity (MFI) analysis of CD86 expression and CXCR4 expression on $CD11b^{+}IgA^{+}$ PP B cells and $CD11b^{-}IgA^{+}$ PP B cells. Bar graphs show the mean values (±SD) of five independent measurements with WT mice**.** Data were compared by two-tailed unpaired Student's t test.

Figure S2

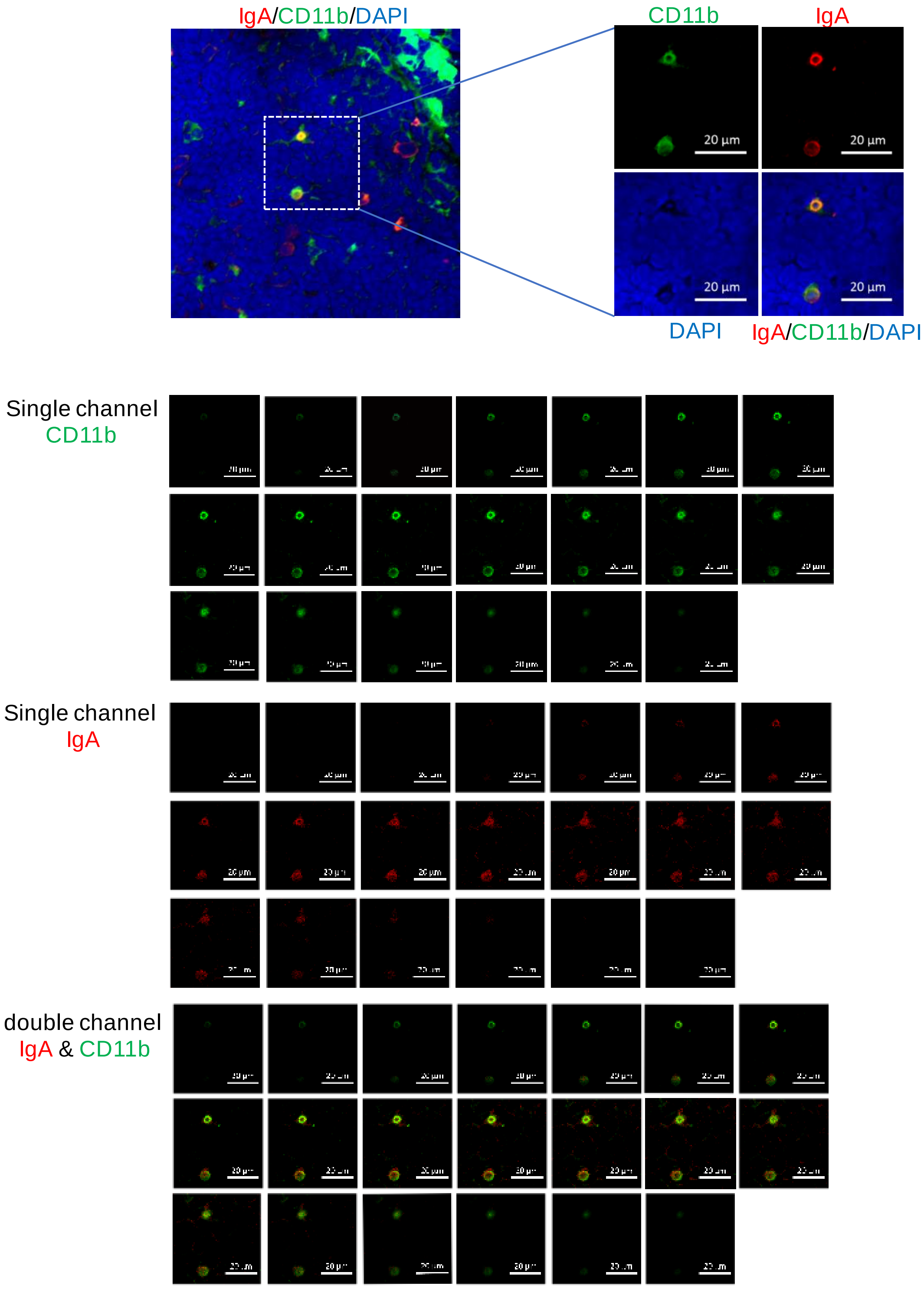

Extended Data Fig. 2. The expressions of CD11b and IgA were confirmed by immunohistochemical confocal imaging.

A selected PP section was stained with DAPI (blue), CD11b (green) and IgA (red). The indicated area (dashed square) was amplified to analyze the overlap of CD11b and IgA. Z-stack analysis was also performed to confirm that CD11b (green) and IgA (red) signals overlapped. Scale bar, 20 μm.

Figure S3

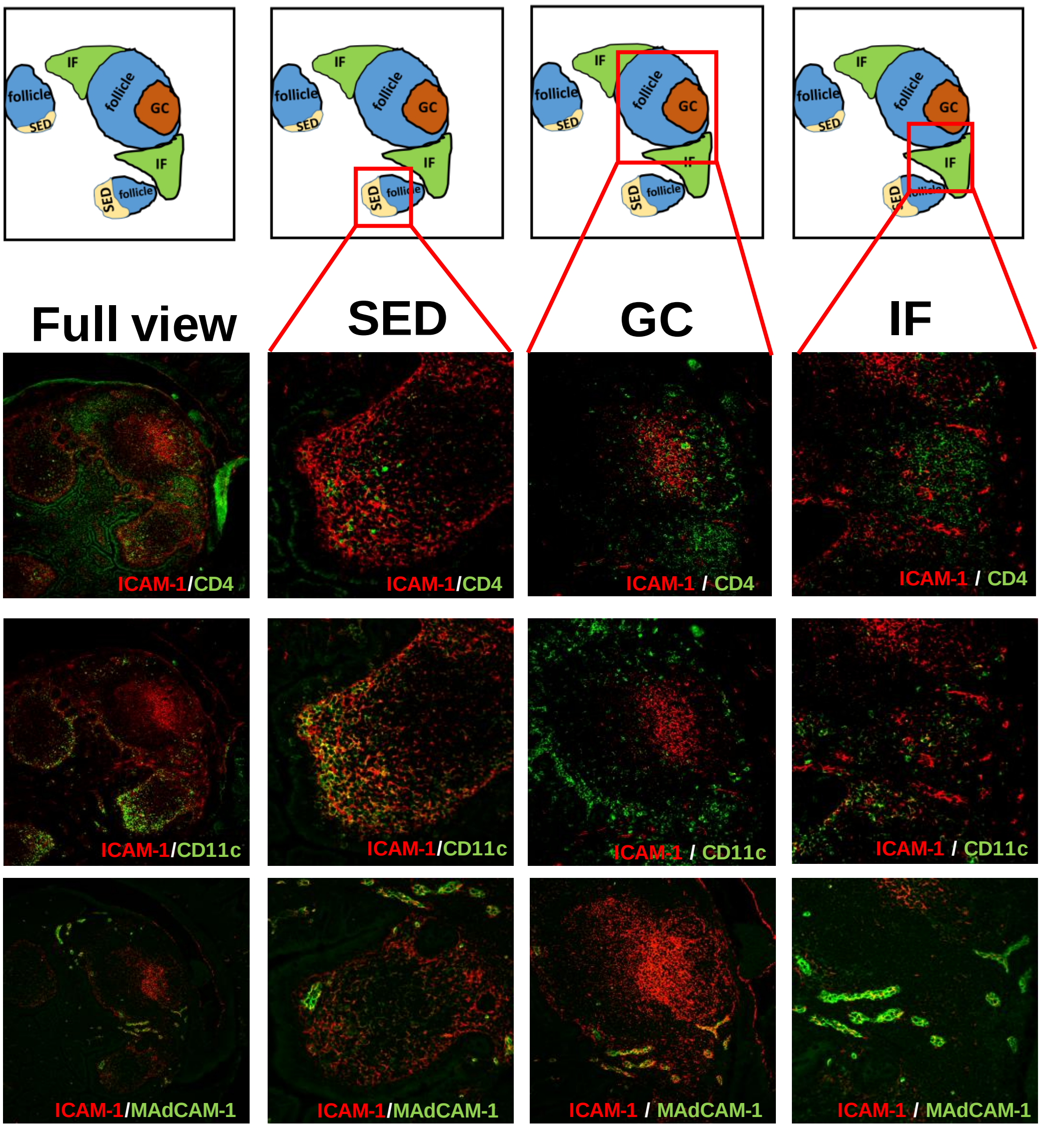

Extended Data Fig. 3. ICAM-1 is expressed by HEVs.

The section of PPs were stained with anti-ICAM-1(red) with anti-CD4 (T cell marker), anti-CD11c (DC marker) or anti-MAdCAM-1 (HEV marker), respectively. SED was identified by CD11c^{+} DC-enriched area. IF area was identified by CD4^{+} T

cell-enriched area. MAdCAM-1-expressing HEVs were found in IF area. Full view and amplified indicated area were shown to analyze ICAM-1 expression.

Figure S4

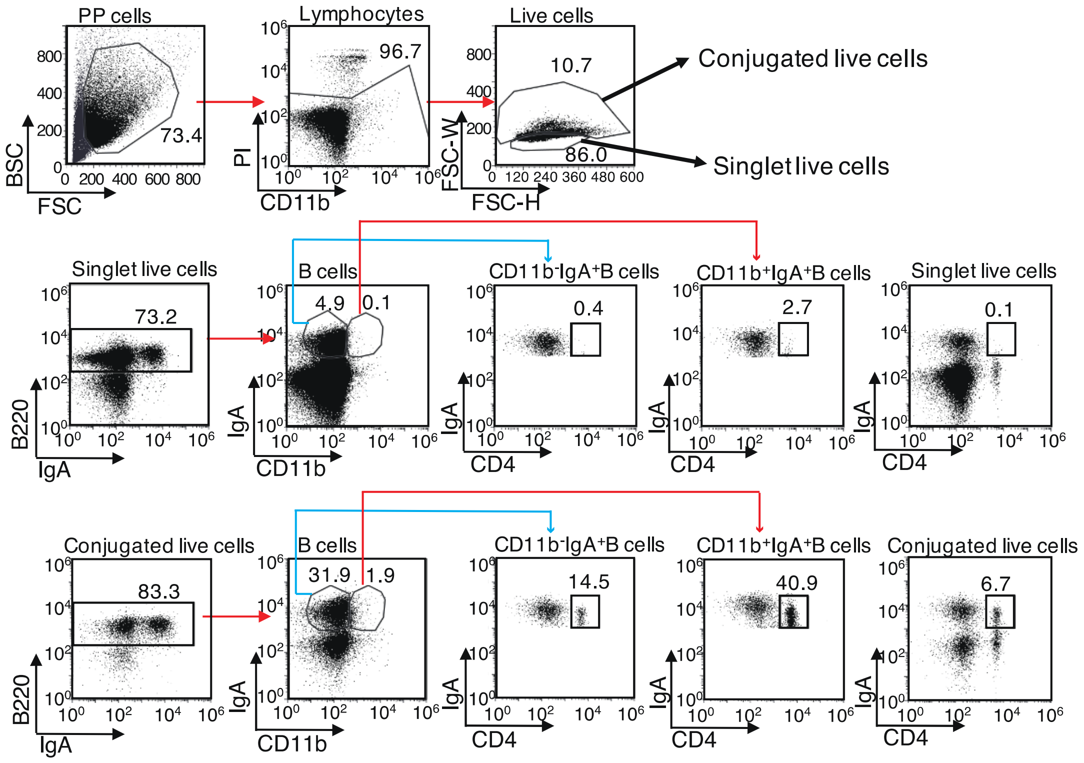

Extended Data Fig. 4. Conjugate analysis of CD11b$^+$IgA$^+$ B cells and CD4$^+$ T cells.

Flow cytometry analysis was performed with WT Balb/c mice of 8-12-week-old. Dead cells were excluded by PI staining. Singlet cells and doublet cells were selected by FSC-H/FSC-W gate. In singlet and conjugate live cells, IgA$^+$ cells were used to analyzed the conjugation with CD4$^+$ T cells. In singlet live cells and conjugate live cells, B cells gate was selected by B220$^+$ cells. IgA and CD11b expressions analyzed

in the $B220^+$ B cells. In conjugate $CD11b^-IgA^+$ B cells and $CD11b^+IgA^+$ B cells, $CD4^+$ T cells conjugation were analyzed. Singlet $CD11b^-IgA^+$ B cells and $CD11b^+IgA^+$ B cells were used as negative controls to analyze the $CD4^+$ T cells conjugation. Data are from 6 independent experiments.

Figure S5

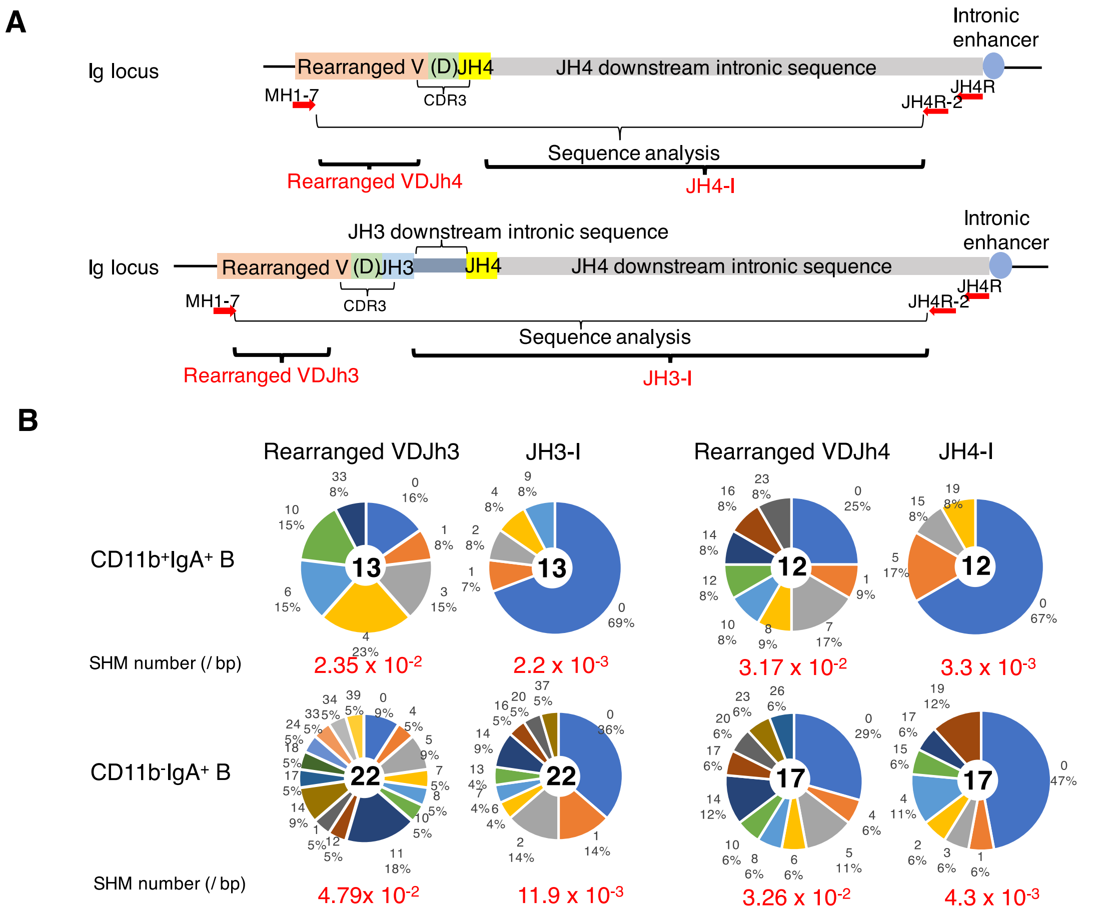

Extended Data Fig. 5. CD11b$^+$IgA$^+$ B cells carrying SHM are derived from newly activated memory B cells.

(A) The rearranged VDJ region and downstream intronic sequences amplified by PCR were analyzed for SHM frequency. **(B)** Analysis of SHM frequency of the rearranged VDJH3 and JH3 downstream intronic sequences (JH3-I) and rearranged VDJH4 and JH4 downstream intronic sequences (JH4-I) of sorted CD11b$^-$IgA$^+$ and CD11b$^+$IgA$^+$ B cells from PPs of 26 WT mice. Segment sizes in pie charts are proportional to the

number of sequences with the mutation numbers observed in each clone. The numbers in the middle of the pie charts represent the numbers of analyzed clones.

Figure S6

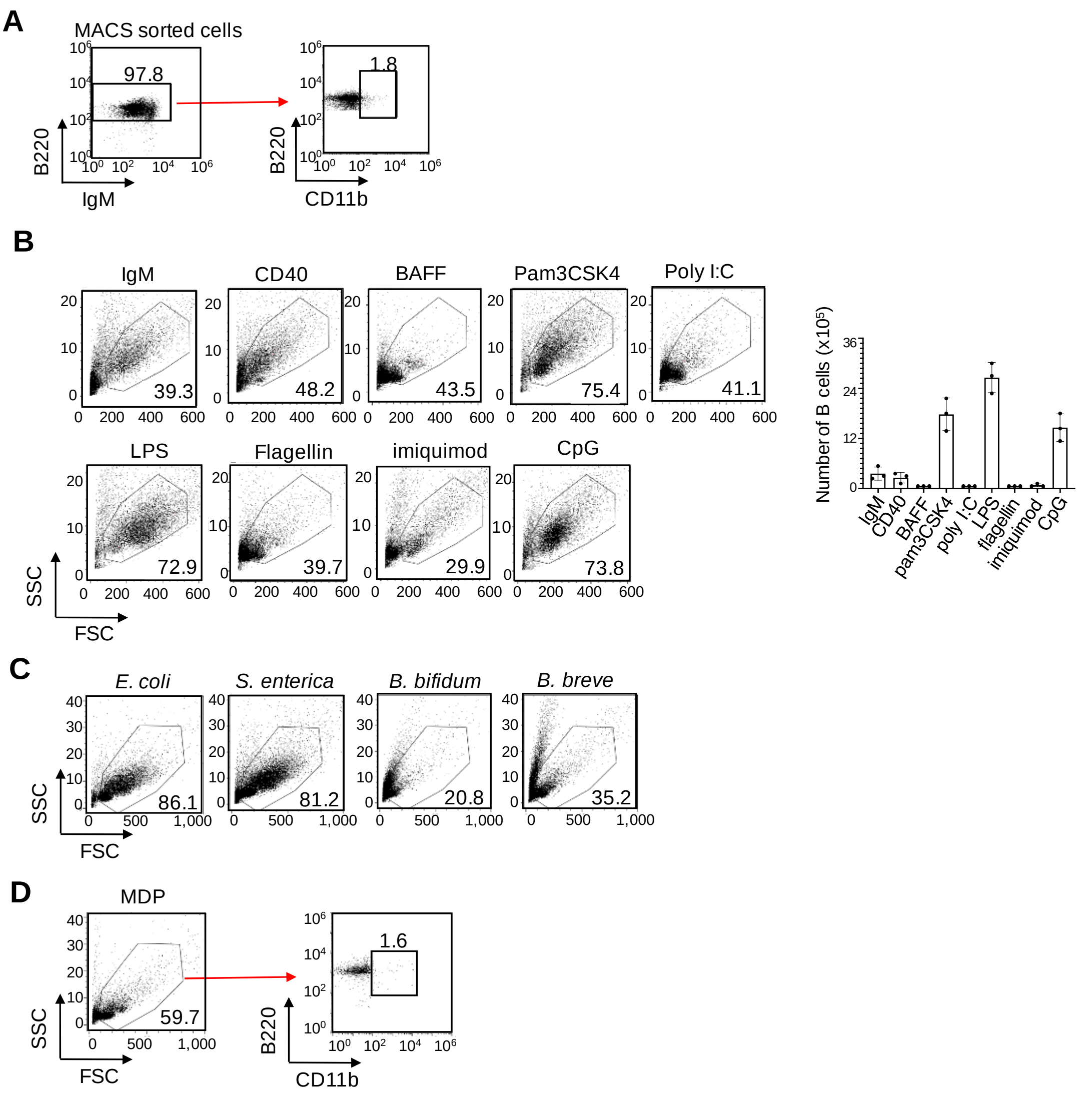
A
MACS sorted cells
97.8
1.8
B220
IgM
CD11b
B
IgM
CD40
BAFF
Pam3CSK4
Poly I:C
39.3
48.2
43.5
75.4
41.1
LPS
Flagellin
imiquimod
CpG
72.9
39.7
29.9
73.8
SSC
FSC
Number of B cells (x10⁵)
IgM
CD40
BAFF
pam3CSK4
poly I:C
LPS
flagellin
imiquimod
CpG
C
E. coli
S. enterica
B. bifidum
B. breve
86.1
81.2
20.8
35.2
SSC
FSC
D
MDP
59.7
1.6
SSC
FSC
B220
CD11b

E

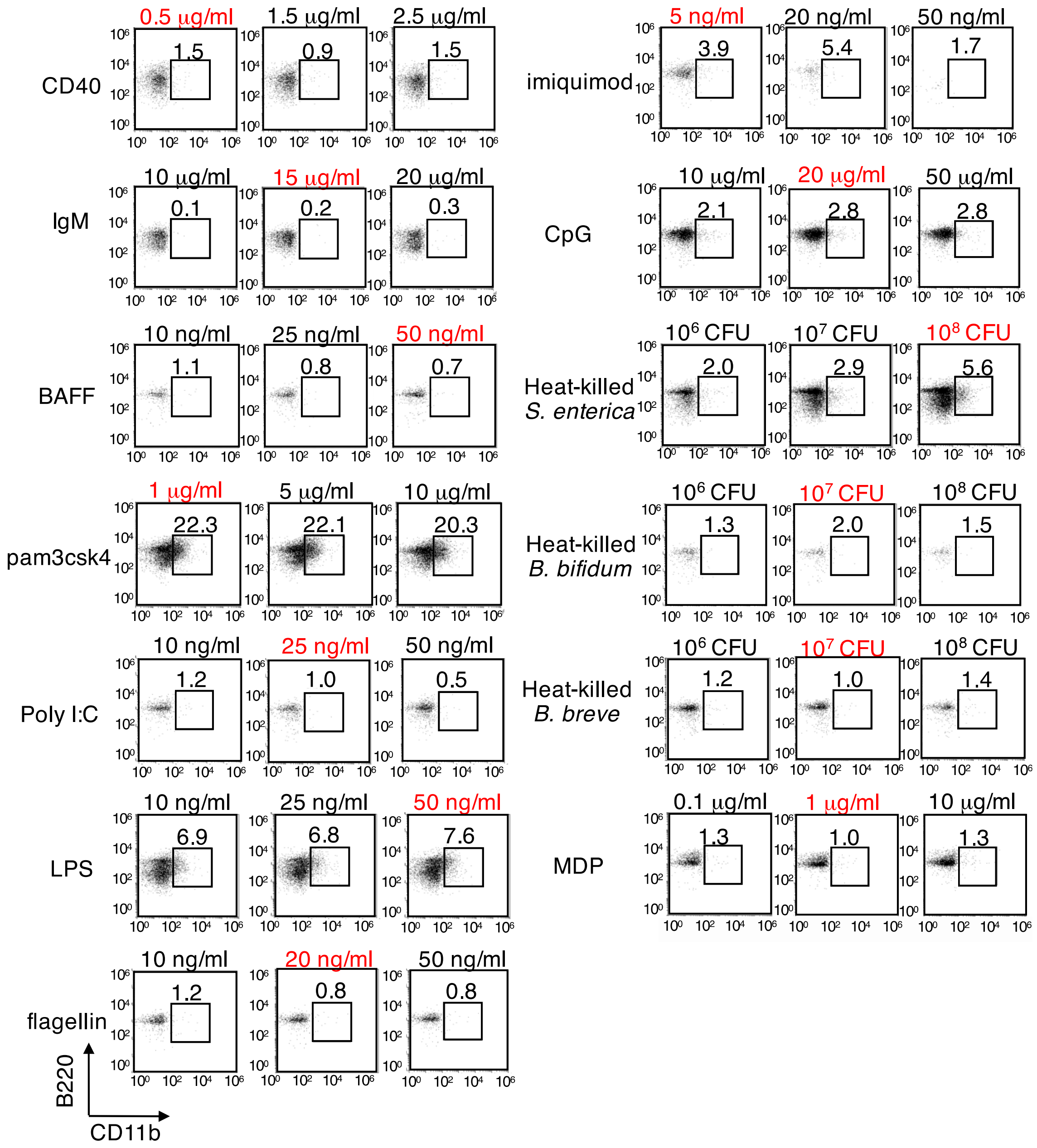

CD40
0.5 μg/ml
1.5 μg/ml
2.5 μg/ml
1.5
0.9
1.5
imiquimod
5 ng/ml
20 ng/ml
50 ng/ml
3.9
5.4
1.7
IgM
10 μg/ml
15 μg/ml
20 μg/ml
0.1
0.2
0.3
CpG
10 μg/ml
20 μg/ml
50 μg/ml
2.1
2.8
2.8
BAFF
10 ng/ml
25 ng/ml
50 ng/ml
1.1
0.8
0.7
Heat-killed S. enterica
10⁶ CFU
10⁷ CFU
10⁸ CFU
2.0
2.9
5.6
pam3csk4
1 μg/ml
5 μg/ml
10 μg/ml
22.3
22.1
20.3
Heat-killed B. bifidum
10⁶ CFU
10⁷ CFU
10⁸ CFU
1.3
2.0
1.5
Poly I:C
10 ng/ml
25 ng/ml
50 ng/ml
1.2
1.0
0.5
Heat-killed B. breve
10⁶ CFU
10⁷ CFU
10⁸ CFU
1.2
1.0
1.4
LPS
10 ng/ml
25 ng/ml
50 ng/ml
6.9
6.8
7.6
MDP
0.1 μg/ml
1 μg/ml
10 μg/ml
1.3
1.0
1.3
flagellin
10 ng/ml
20 ng/ml
50 ng/ml
1.2
0.8
0.8
B220
CD11b

F

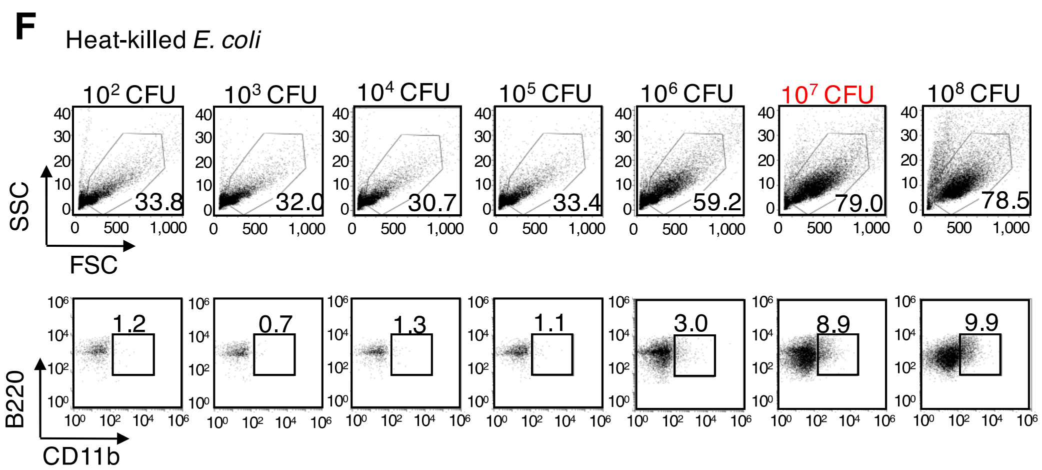

G

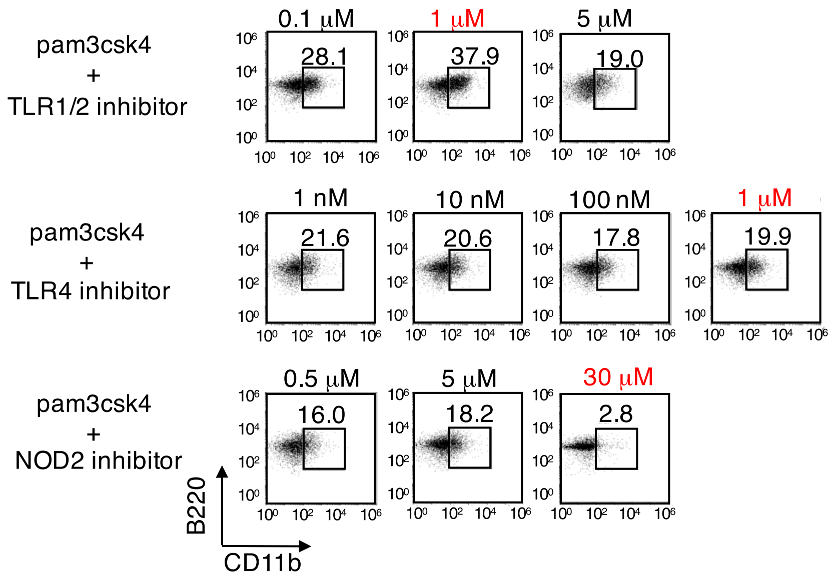

Extended Data Fig. 6. B cell proliferation and CD11b expression by *in vitro* stimulation.

(A) The purity of sorted naïve spleen B cells and their CD11b expression. (**B**) Lymphocyte gate and numbers (right) of the cultured spleen naïve B cells with indicated single stimulations. Representative data are from at least three independent

experiments. (**C**) Lymphocyte gate of the cultured spleen naïve B cells with indicated heat-killed bacteria stimulations. (**D**) Lymphocyte gate and CD11b expression of the cultured spleen naïve B cells with MDP. Representative data are from three independent experiments. (**E, F**) CD11b expression of purified spleen B cells stimulated with indicated concentration of each stimulation. Selected conditions for replicated experiments are marked in red color. **(G)** CD11b expression of pam3CSK4 (1 μg/ml)-stimulated spleen B cells in the presence of indicated concentration of TLR1/2, TLR4 and NOD2 inhibitors. Selected conditions for replicated experiments are marked in red color

Figure S7

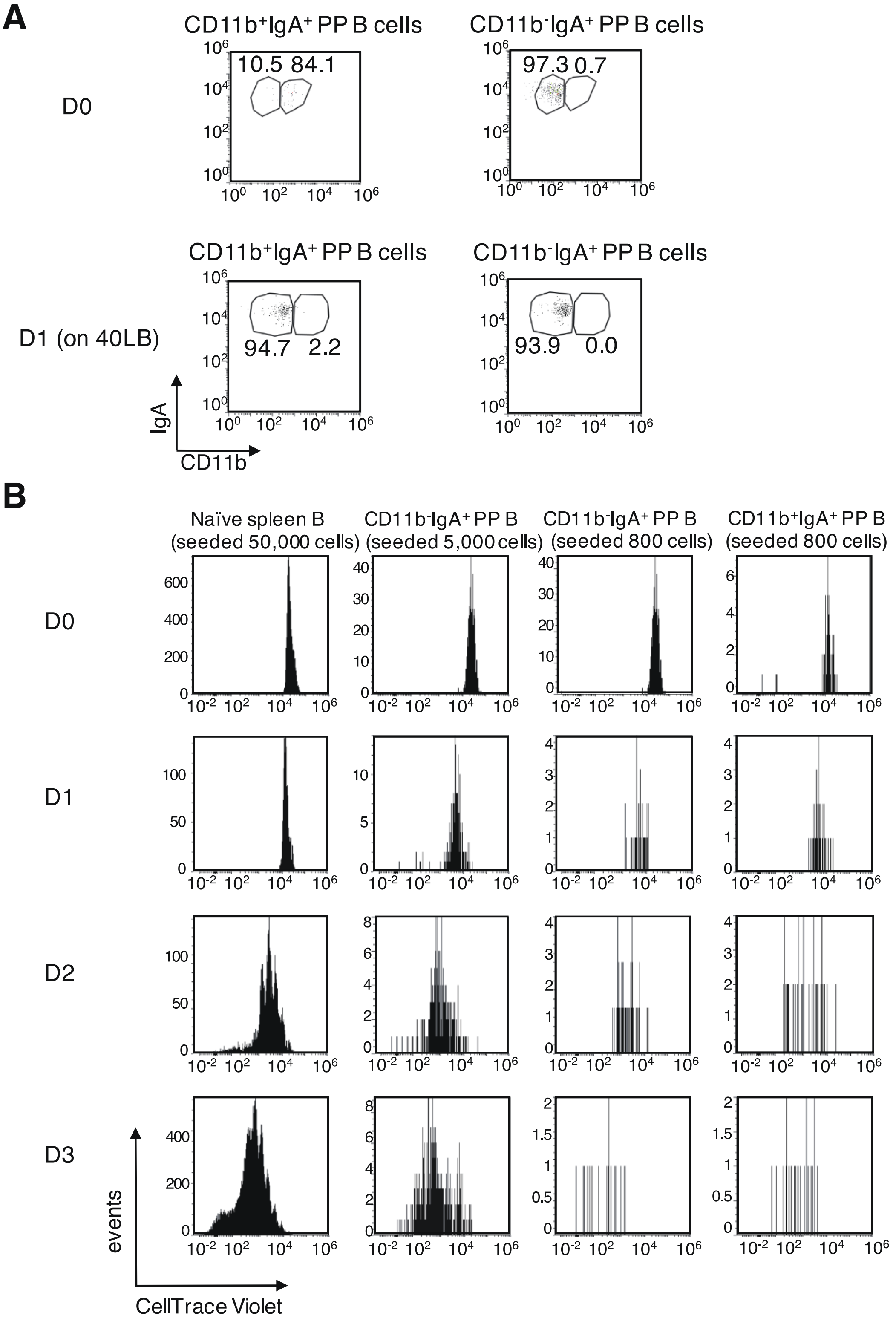
A
CD11b+IgA+ PP B cells
CD11b-IgA+ PP B cells
D0
10.5 84.1
97.3 0.7
D1 (on 40LB)
94.7 2.2
93.9 0.0
IgA
CD11b
B
Naïve spleen B (seeded 50,000 cells)
CD11b-IgA+ PP B (seeded 5,000 cells)
CD11b-IgA+ PP B (seeded 800 cells)
CD11b+IgA+ PP B (seeded 800 cells)
D0
D1
D2
D3
events
CellTrace Violet

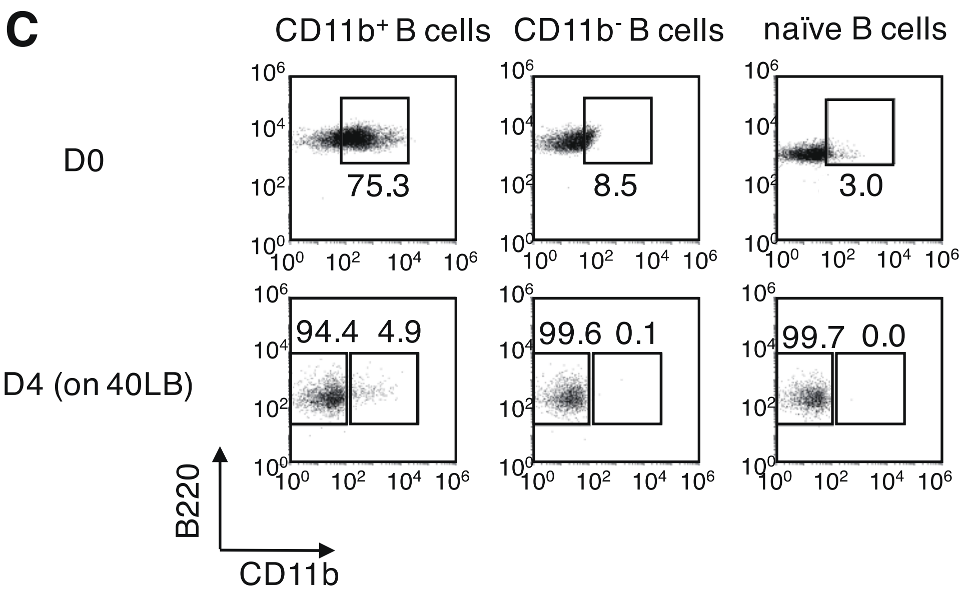

Extended Data Fig. 7. Sorted CD11b^{+} B cells lost their CD11b expression on iGB culture system.

(A) Sorted CD11b^{+}IgA^{+} and CD11b^{-}IgA^{+} PP B cells labeled with CellTrace Violet were cultured on iGB culture system with IL-21. CD11b expression was analyzed by flow cytometry on day 0 and day 1. (**B**) About 50,000 naïve spleen B cells and 5,000 sorted CD11b^{-}IgA^{+} B cells labelled with CellTrace Violet were prepared as positive controls. Eight-hundred sorted CD11b^{+}IgA^{+} PP B cells and CD11b^{-}IgA^{+} PP B cells were prepared to monitor their proliferation. Representative data are shown from two independent experiments. (**C**) Spleen naïve B cells were cultured with pam3CSK4 for 3 days to induce CD11b^{+} B cells. Then, the pam3CSK4-induced CD11b^{+} and CD11b^{-} B cells were sorted. About 1.5 x 10^{5} sorted CD11b^{+} B cells and CD11b^{-} B cells were

seeded on 40LB cells with 1 ng/ml IL-4, separately. Negatively sorted spleen naïve B cells were used as controls. The CD11b expression of indicated B cells seeded on 40LB system were analyzed on D0 and D4. Representative data are shown from three independent experiments.

Figure S8

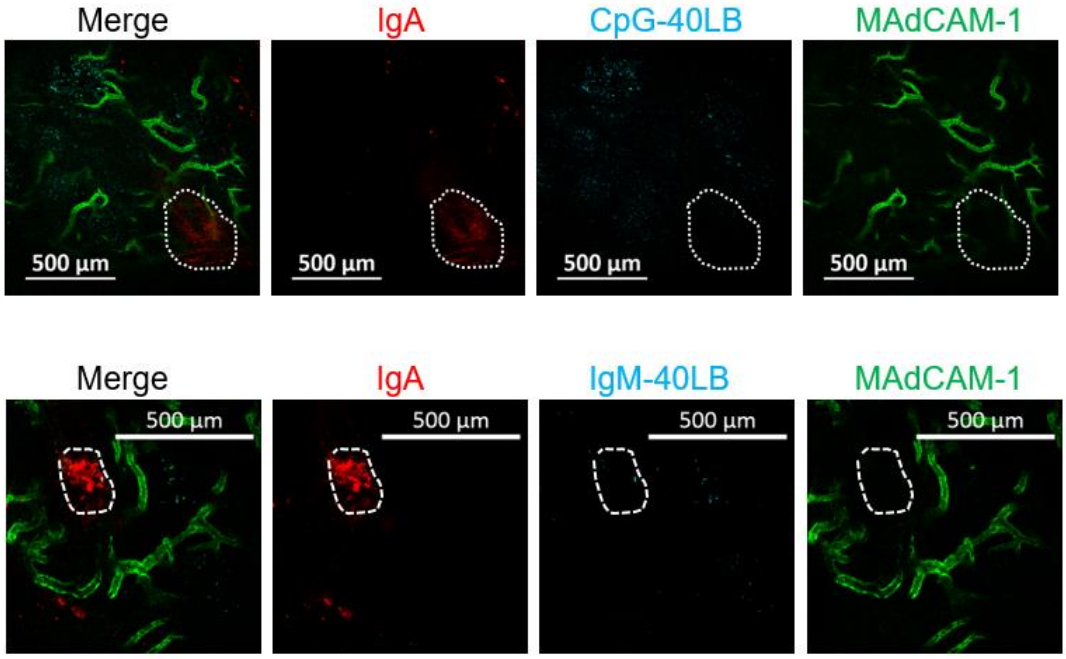

Extended Data Fig. 8. B cells stimulated with IgM-40LB or CpG-40LB did not enter PP GC after intravenous injection to mice.

Indicated iGB cells, including CpG-40LB cells (up) and IgM-40LB cells (down) were sorted, labeled (cyan) and then intravenously injected to mice, independently. With the labeled iGB cells, anti-MAdCAM-1 antibody was injected intravenously to identify the HEVs (green). Anti-IgA antibody was directly injected to a PP to stain the IgA^+ cells (red) for GC (dashed circle) identification. Scale bar, 500 μm.
